# Utilization of Wild Edible Plants by the Tai Yoy Ethnic Group in Akat Amnuai District, Sakon Nakhon Province, Thailand

**DOI:** 10.3390/biology15010015

**Published:** 2025-12-20

**Authors:** Piyaporn Saensouk, Surapon Saensouk, Sombat Appamaraka, Kamonwan Koompoot, Anousone Sengthong, Kajonesuk Phengmala, Tammanoon Jitpromma

**Affiliations:** 1 Diversity of Family Zingiberaceae and Vascular Plant for Its Applications Research Unit, Mahasarakham University, Kantarawichai District, Kantarawichai District, Maha Sarakham 44150, Thailand; pcornukaempferia@yahoo.com (P.S.); sombat_amp@yahoo.co.th (S.A.); kamonwan.k@kkumail.com (K.K.); jitpromma.t@gmail.com (T.J.); 2 Department of Biology, Faculty of Science, Mahasarakham University, Kantarawichai District, Maha Sarakham 44150, Thailand; 3 Walai Rukhavej Botanical Research Institute, Mahasarakham University, Kantarawichai District, Maha Sarakham 44150, Thailand; 4 Faculty of Forest Science, National University of Laos, Vientiane 7322, Laos; a.sengthong@nuol.edu.la (A.S.); k.phengmala@nuol.edu.la (K.P.)

**Keywords:** Akat Amnuai district, ethnobotany, ethnomedicine, medicinal plants, Sakon Nakhon, Tai Yoy ethnic group, wild edible plants (WEPs)

## Abstract

Wild edible plants (WEPs) play a crucial role in sustaining local food systems, cultural heritage, and livelihoods in northeastern Thailand. This study documents the diversity, cultural significance, medicinal use, and economic value of WEPs among the Tai Yoy community in Akat Amnuai District, Sakon Nakhon Province. Field surveys, interviews, and market observations recorded 78 species belonging to 42 families, with 52 species represent new records for the province. Fruits and leaves were the most commonly used parts, while multifunctional species such as *Curcuma angustifolia* Roxb., *Phyllanthus androgynus* (L.) Chakrab. & N.P.Balakr., and *Spondias pinnata* (L.f.) Kurz were highly valued for both dietary and medicinal purposes. Quantitative analyses, including the Cultural Food Significance Index (CFSI), fidelity level (FL), and Jaccard’s similarity index (JI), revealed strong cultural consensus on key species and regional patterns shaped by ecological and sociocultural factors. Market surveys identified 25 species contributing to household income, highlighting the economic importance of WEPs and the potential risks of overharvesting. The study emphasizes the need for sustainable management, including cultivation of high-demand species, to reduce pressure on wild populations while supporting food security and traditional knowledge. These findings provide a comprehensive baseline for future ethnobotanical research, conservation planning, and the sustainable use of WEPs in northeastern Thailand.

## Simple Summary

Wild edible plants are important sources of food, medicine, and income for many rural communities, but their use is often overlooked and not well documented. This study explored how the Tai Yoy community in northeastern Thailand uses wild plants for food and traditional medicine. A total of 78 species were recorded, including 52 plants that had not been previously reported in the region. Fruits and leaves were the most commonly eaten parts, while some plants also provided medicinal benefits or could be sold in local markets, contributing to household income. The study found that certain species are highly valued and widely used, showing strong agreement among community members about their usefulness. However, many of these plants are collected from forests and fallow lands, which could lead to overharvesting if not managed carefully. Encouraging the cultivation of high-demand species in home gardens or community plots could reduce pressure on wild populations, ensure year-round availability, and help maintain traditional knowledge. By documenting how these plants are used, this research supports the conservation of plant diversity, promotes sustainable use of natural resources, and highlights the important role of wild edible plants in local food security, health, and cultural heritage.

## 1. Introduction

Wild edible plants (WEPs) have been integral to human diets for millennia, providing not only essential nutrients but also serving as sources of medicine, flavoring, and cultural symbolism. Across rural and indigenous communities worldwide, these plants contribute significantly to food security, especially during periods of scarcity or seasonal variation in cultivated crops [1]. In addition to nutritional benefits, WEPs are closely linked to traditional ecological knowledge (TEK), reflecting a community’s understanding of local biodiversity, resource management, and environmental stewardship [2]. Ethnobotanical documentation of these practices is therefore crucial not only for preserving indigenous knowledge but also for informing sustainable agriculture, conservation, and potential nutraceutical development [3].

Southeast Asia is recognized as a center of biodiversity, with a rich array of plant species adapted to diverse ecological niches. In this region, traditional knowledge regarding WEPs is highly developed, forming an essential component of rural livelihoods and cultural identity [4,5,6]. Communities across Thailand rely on forest, riverine, and agricultural ecosystems to supplement their diets, obtaining leafy vegetables, fruits, roots, seeds, and other plant products from wild or semi-wild sources [7]. These resources are not only consumed for sustenance but also incorporated into local culinary traditions, rituals, and medicinal practices. Studies in neighboring provinces and ethnic groups have highlighted the diversity of species utilized, patterns of seasonal availability, and the role of gender, age, and cultural norms in knowledge transmission [8].

Although several ethnobotanical studies in Thailand have been conducted among major ethnic groups, most of them have focused on communities in the northeastern and northern regions [7,9]. However, the Tai Yoy ethnic group in Sakon Nakhon Province has received very limited scholarly attention. To date, only a single study has investigated medicinal plants used by the Tai Yoy, and it was restricted to a single community forest area [10]. The Tai Yoy have maintained a distinct cultural and linguistic identity for generations and have relied heavily on their surrounding natural environment for food, medicine, and cultural practices. Their knowledge of WEPs encompasses species identification, seasonal availability, harvesting techniques, preparation methods, and associated cultural beliefs. Such practices reflect a deep understanding of ecological dynamics and sustainable resource use [11]. Nevertheless, systematic documentation of the Tai Yoy’s ethnobotanical knowledge remains scarce, leaving a significant gap in both regional and national records of plant utilization.

Akat Amnuai District (AAD) in Sakon Nakhon Province represents a landscape where forests, agricultural fields, and village gardens coexist, offering a diverse array of WEP species [12]. The district is home to several Tai Yoy communities, whose subsistence strategies continue to incorporate wild plants alongside cultivated crops. As modernization and land-use changes advance, traditional knowledge of wild plant utilization faces threats from cultural erosion, habitat loss, and changing dietary habits [13]. Preservation of this knowledge is therefore urgent, not only for cultural heritage but also for sustainable food security and biodiversity conservation [14].

The present study aims to document the diversity, utilization, and cultural significance of WEPs among the Tai Yoy ethnic group in AAD. Specifically, it seeks to (i) inventory the WEP species used by the community, (ii) describe their various uses, including culinary, medicinal, and other applications, (iii) assess the cultural significance of these plants within the community, and (iv) compare the species composition and use of WEPs in AAD with selected surrounding areas using the Jaccard Similarity Index (JI). By generating a comprehensive ethnobotanical record, this research provides a foundation for further studies on nutritional potential, sustainable resource management, and conservation planning. In addition, the study contributes to broader efforts to recognize and preserve the TEK of Thailand’s ethnic minorities, highlighting the interconnection between culture, biodiversity, and food systems.

## 2. Materials and Methods

### 2.1. Study Area Description

**Figure 1:**
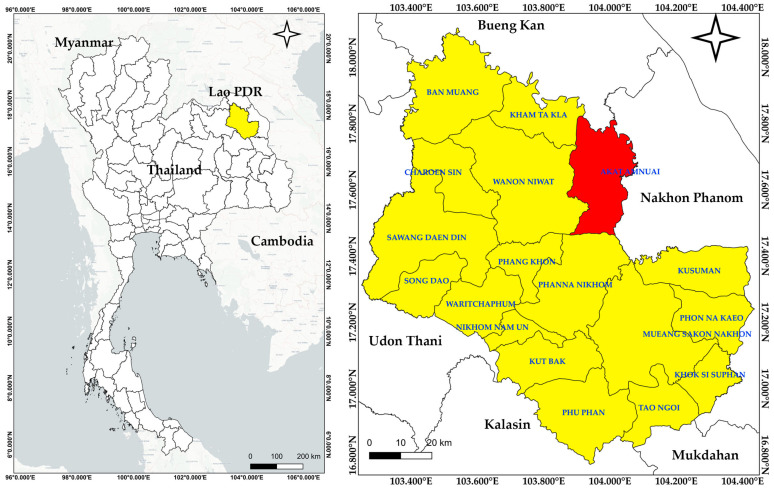
Map of the study area: Left shows Sakon Nakhon Province in yellow within Thailand. The right highlights Akat Amnuai District in red within the province (map created with “QGIS” program ver. 3.34 [15], geographic system ID: WGS 84, EPSG 4326).

This study was conducted in AAD, located in the northern part of Sakon Nakhon Province, Northeastern Thailand (Figure 1). The district is divided into eight sub-districts and covers a total area of approximately 585 km^2^. Geographically, it lies between 17°32′–17°43′ N latitude and 104°05′–104°22′ E longitude, with the central coordinates at 17°35′48″ N, 103°58′30″ E, and an elevation of approximately 509 ft (155 m) above sea level. AAD is bordered by Seka District of Bueng Kan Province to the north; Na Thom, Si Songkhram, and Na Wa Districts of Nakhon Phanom Province to the east; and Phanna Nikhom, Wanon Niwat, and Kham Ta Kla Districts of Sakon Nakhon Province to the south and west.

The area experiences consistently warm temperatures throughout the year, with daily averages ranging from approximately 16 °C (60 °F) to 34 °C (94 °F). The hot season occurs from late March to mid-May, with average daily temperatures exceeding 33 °C (91 °F), peaking in April at around 34 °C (93 °F). The cool season occurs from late November to February, with average daily temperatures below 29 °C (84 °F) and December being the coolest month, with average lows around 17 °C (62 °F) and highs around 28 °C (82 °F). The rainy season generally spans late April to early October, with days receiving more than 0.04 inches of rain on over 39% of days, peaking in August with an average of 23 rainy days. In contrast, the dry season lasts approximately 6.8 months, from early October to late February, with December having the fewest rainy days, averaging only 0.5 days [16].

The local inhabitants are predominantly of the Tai Yoy ethnic group, who maintain strong cultural traditions, particularly in their relationship with nature and the use of local plant resources. Subsistence farming, including rice cultivation, vegetable gardening, and foraging for wild plants, forms an integral part of their livelihood [10]. The selected study villages within AAD were chosen based on the presence of Thai Yoy communities with well-preserved TEK and frequent utilization of WEP species.

### 2.2. Plant Collection and Identification

All WEP species mentioned by informants were collected during field walks and market visits. Specimens were photographed in the field, prepared as herbarium vouchers, and deposited in the Vascular Plant Herbarium, Mahasarakham University (VMSU) in Kantharawichai District, Maha Sarakham Province, Thailand, for long-term storage. Taxonomic identification of the collected plant species was carried out using standard floras, including Flora of Thailand, as well as relevant scientific publications. The scientific names and family classifications were further verified through the online database Plants of the World Online (POWO) [17].

### 2.3. Local Market Survey

To document the utilization of WEPs by the Thai Yoy ethnic group, a systematic survey of local markets in AAD was conducted from December 2024 to November 2025. The survey focused on markets frequented by local residents, where WEPs are commonly sold or displayed, including fresh produce, herbs, spices, and processed products.

Data collection involved direct observation, structured interviews with market vendors and local collectors, and photographic documentation of plant specimens. For each plant species recorded, information was gathered on the local name, plant part used, form of sale (fresh, dried, or processed), seasonal availability, and reported uses. In addition, data on selling price and the quantity sold per month were collected to assess the economic value of each species to local livelihoods.

The survey was conducted monthly to capture seasonal variation in both the availability and market dynamics of WEPs. All information was recorded in pre-designed data sheets, and the identities of informants were anonymized to ensure privacy and ethical compliance.

This approach allowed for a comprehensive assessment of the diversity, cultural importance, market utilization, and economic contribution of WEPs among the Thai Yoy community.

### 2.4. Ethnobotanical Data Collection

Ethnobotanical fieldwork was conducted from December 2024 to November 2025 across eight sub-districts predominantly inhabited by the Tai Yoy ethnic group. The study employed qualitative ethnobotanical approaches, combining semi-structured interviews, participant observation, and guided field walks with experienced local informants.

A total of 40 informants (20 men and 20 women, aged 25–65 years) were selected through snowball [18] and purposive sampling techniques [19], prioritizing traditional healers, elderly villagers, and individuals widely recognized for their plant knowledge. Interviews were carried out in Thai or the local Yoy dialect, focusing on topics such as local plant names, parts used, preparation and consumption methods, collection sites, seasonal availability, and the cultural or symbolic importance of each species.

Before data collection, the objectives and procedures of the study were clearly explained to all participants, and prior informed consent was obtained in full compliance with the International Society of Ethnobiology (ISE) Code of Ethics [20] and the Nagoya Protocol on Access and Benefit-Sharing [21]. Participants were informed of their rights, including voluntary participation and the ability to withdraw at any stage without consequence.

Although the research did not involve collecting personal or sensitive information and therefore did not require formal institutional ethical approval, it strictly followed international ethical standards for ethnobotanical research to ensure respect, transparency, and reciprocity toward the participating communities.

### 2.5. Categorization of Plant Uses

Each recorded plant species was categorized according to its principal mode of utilization, following the framework of Saensouk et al. [22] with slight modifications for this study. The categories included condiments and flavoring, fruits, staple foods, sweets, desserts and snacks, vegetables, and medicinal edible plants.

These categories are not entirely homogeneous, reflecting the cultural and culinary complexity of Thai foodways. In Thai cuisine, a single plant may be incorporated into multiple dishes, prepared in diverse ways (raw, boiled, fermented, roasted, or pickled), or serve multiple roles within a meal—such as functioning simultaneously as a vegetable, seasoning, or medicinal food. Consequently, some species could legitimately fit more than one category. To account for this, species appearing in multiple food-related subcategories were counted only once in the broader “food” domain during quantitative analysis; subcategories were used solely to illustrate culinary diversity and cultural context.

Certain plant parts that are botanically fruits, such as young pods or bitter fruits, are commonly used as vegetables in Thai cuisine. For example, the fruits of *Oroxylum indicum* are consumed as a vegetable, typically served with chili paste, reflecting the distinction between botanical classification and culinary practice in Thailand, where “fruits” generally refer to sweet-tasting produce.

### 2.6. Data Analysis

#### 2.6.1. Cultural Food Significance Index (CFSI)

In this study, the Cultural Food Significance Index (CFSI) was employed to evaluate the cultural importance of WEPs used by the Tai Yoy ethnic group for both dietary and medicinal purposes. The index, originally proposed by Pieroni [23], integrates seven ethnobotanical indicators into a single quantitative measure of a species’ cultural value, expressed as

CFSI = QI × AI × FUI × PUI × MFFI × TSAI × FMRI × 10^−2^ (1)

The Quotation Index (QI) represents the proportion of informants who mentioned a particular species. The Availability Index (AI) reflects the local abundance of the species, ranging from very common (4.0) to rare (1.0). The Frequency of Utilization Index (FUI) quantifies how often the species is consumed, with values from more than once per week (5.0) to no longer used within the past 30 years (0.5). The Parts Used Index (PUI) measures the diversity of plant parts utilized, such as roots, stems, leaves, flowers, fruits, or seeds. The Multifunctional Food Use Index (MFFI) evaluates the range of culinary applications, including methods like raw consumption, boiling, frying, roasting, or use as condiments. The Taste Score Appreciation Index (TSAI) assesses local perceptions of flavor and palatability, from terrible (4.0) to excellent (10.0). Finally, the Food-Medicinal Role Index (FMRI) captures the degree to which a plant is recognized as both a food and a medicinal resource, ranging from not recognized (1.0) to very high (5.0).

#### 2.6.2. Cluster Analysis

To examine and visualize variation in Cultural Food Significance Index (CFSI) values among WEP species recorded in the study area, the Unweighted Pair Group Method with Arithmetic Mean (UPGMA) [24] was employed. The data matrix of CFSI values was analyzed using the UPGMA clustering algorithm, and the relationships among species were represented as a heatmap generated with PAST4 software (version 4.15). This analytical approach enabled a comparative evaluation of CFSI patterns, providing insights into the cultural importance, diversity, and utilization patterns of WEPs within the Tai Yoy community.

#### 2.6.3. Informant Consensus Factor (F_ic_)

The Informant Consensus Factor (F_ic_) was employed to assess the degree of agreement among informants regarding the use of WEPs for specific medicinal purposes. This measure helps identify ailment categories for which there is strong collective knowledge and consistent plant use within the community. F_ic_ was calculated following Heinrich et al. [25] using the equation

(2) $$F_{\mathrm{ic}}=\frac{n_{\mathrm{ur}}-n_{t}}{n_{\mathrm{ur}}-1}$$

where n_ur_ represents the total number of use reports in each medicinal category, and n_t_ denotes the number of species cited in that category. F_ic_ values range from 0 to 1, with values approaching 1 indicating a high level of consensus among informants. Such high values suggest that certain ailments are well recognized and that specific plant remedies are widely shared and trusted within the community.

#### 2.6.4. Fidelity Level (%FL)

The Fidelity Level (FL) was used to determine the degree to which a given plant species is specifically associated with a particular medicinal use. This index reflects the proportion of informants who cited a species for the same therapeutic purpose, providing insight into the cultural importance and reliability of traditional plant-based remedies. FL was calculated as described by Friedman et al. [26] using the formula

(3) $$\mathrm{FL}=\frac{I_{p}}{I_{u}}\;\times \;100$$

where I_p_ is the number of informants who cited a species for a specific ailment, and I_u_ is the total number of informants who mentioned that species for any medicinal use. Higher FL values indicate that most informants consistently associate the species with a single, well-defined therapeutic use, reflecting strong cultural agreement and specificity in ethnomedicinal knowledge.

#### 2.6.5. WEPs Comparison with Surrounding Areas (Jaccard Index)

To compare species composition and assess the similarity of WEPs use between the current study area and neighboring surrounding areas, previously published data from ethnobotanical studies were reviewed and species composition data were extracted. Scientific names of all species were verified and updated to their current accepted nomenclature before comparison. The Jaccard Similarity Index (JI) was then employed to quantify the proportion of species shared between the current dataset and the published datasets. JI is calculated as follows [27]:

JI = c/(a + b − c)(4)

where a represents the number of species in study area A, b is the number of species in study area B, and c denotes the number of species common to both areas. The JI ranges from 0 (no similarity) to 1 (complete similarity). Multiplying the JI value by 100 converts it into a similarity percentage (%) (SM), providing a more intuitive measure of species overlap between study sites.

#### 2.6.6. Economic Value Assessment

The Economic Value (EV) assessment was undertaken to evaluate the contribution of WEPs to the local economy of the Tai Yoy community in AAD, Sakon Nakhon Province. The analysis integrated quantitative market data with botanical identification to ensure the precise recognition of traded species. Surveys were conducted among local vendors and consumers in community markets, focusing on plant species actively collected, sold, and purchased within the district. Market price data were recorded monthly from December 2024 to November 2025 to capture seasonal variations in availability and sales.

To estimate the annual economic value of each species, the average price (AP) was calculated as the mean of its maximum and minimum market prices. The Economic Value (EV) was then computed using the equation [28]

EV = AP × SM × SP(5)

where EV denotes the annual economic value of each WEP species, AP represents the average price per kilogram (THB/kg), SM indicates the total monthly sales volume (kg), and SP refers to the number of months each species was available for sale throughout the year. This value reflects the total annual market income generated from the trade of WEPs, providing insights into their economic significance and role in sustaining local livelihoods among the Tai Yoy ethnic group.

## 3. Results

### 3.1. Diversity of WEPs

**Figure 2:**
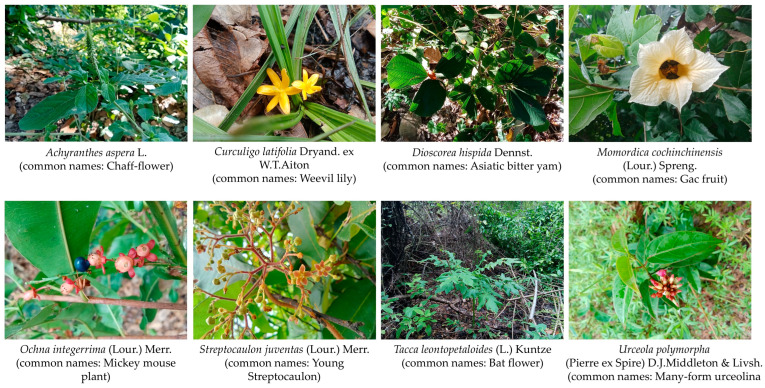
Selected examples of wild edible plant species utilized by the Tai Yoy ethnic group in Akat Amnuai District, Sakon Nakhon Province, Thailand. Photos by Tammanoon Jitpromma.

A total of 78 wild edible plant species belonging to 74 genera and 42 families were documented from the study area (Table 1 and Table 2 and Figure 2). Fabaceae was the most represented family (7 species, 8.97%), followed by Apocynaceae (5 species, 6.41%) and Zingiberaceae (4 species, 5.13%). Several other families were represented by 2–3 species each, while the remaining families were represented by a single species. Table 1 provides the full list of families, number of species, and their relative percentages.

**Figure 3:**
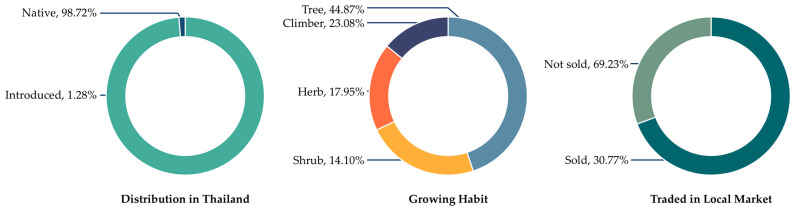
Proportion of distribution in Thailand, growing habit, and trade in local market.

The majority of WEPs documented among the Tai Yoy community were native species, reflecting the community’s strong reliance on locally available biodiversity. Trees constituted the most common growth form, indicating the importance of forest-based resources in daily subsistence. Although most species were gathered exclusively for household consumption, a smaller subset was also traded in local markets, suggesting their dual roles in both food security and livelihood support (Figure 3).

**Figure 4:**
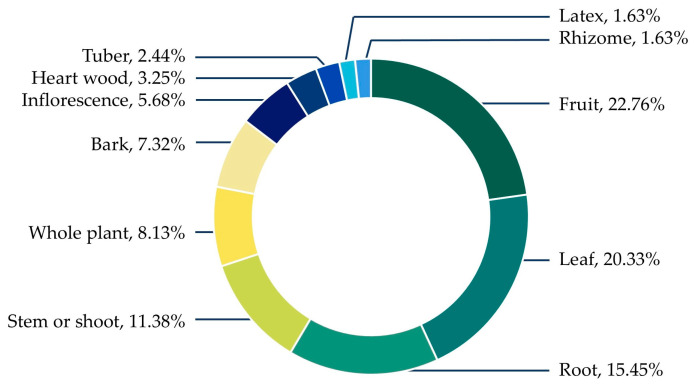
Proportion of plant parts used.

Regarding medicinal applications, fruits and leaves emerged as the dominant plant parts used, highlighting the preference for readily accessible and renewable materials. Roots and shoots were also important, particularly in preparations requiring boiling or decoction, which aligns with traditional medicinal practices. Less frequently used parts—such as bark, inflorescences, heartwood, and tubers—were typically associated with specific therapeutic purposes that require more selective harvesting (Figure 4).

### 3.2. Utilization of WEPs

#### 3.2.1. WEPs Used as Condiments and Flavoring

Several WEPs utilized by the Tai Yoy ethnic group serve as condiments and flavoring agents, playing an important role in enhancing the taste, aroma, and sensory appeal of traditional dishes. A total of seven species belonging to six families were documented in this category (Table 2).

Among them, *Spondias pinnata* is highly valued for its sour–sweet fruits, which are commonly added to various local dishes to enrich flavor. *Piliostigma malabaricum*, *Senegalia rugata*, and *Urceola polymorpha* are notable for their sour fruits and leaves, which are widely used as natural flavor enhancers in everyday cooking. Additionally, *S. pennata* has a secondary traditional use, where its mature fruits are sun-dried and boiled with turmeric to make a herbal hair-washing solution, reflecting its cultural versatility.

Plants from the Hypericaceae family, including *Cratoxylum cochinchinense* and *C. formosum*, are frequently used for their young leaves and inflorescences, which impart a distinct sourness and enhance the flavor of local dishes. Furthermore, *Boesenbergia rotunda* (Zingiberaceae) is a well-known aromatic rhizome, often incorporated into curry pastes and various recipes to add both spice and fragrance, underscoring its culinary and ethnobotanical importance among the Tai Yoy community.

#### 3.2.2. WEPs Used as Fruits

Among the WEPs documented in AAD, a total of 18 species from 15 families were consumed as fruits (Table 2). These species are utilized at different stages of fruit maturity, either unripe or ripe, depending on their taste and traditional culinary practices.

Two species were reported as being consumed when unripe. *Ficus racemosa* is eaten as a fresh vegetable in its unripe stage, while *Mangifera caloneura* is consumed both raw (green) and ripe, reflecting its dual use as a sour-tasting fruit when unripe.

The majority of species (16 species) are eaten ripe, primarily for direct consumption as fresh fruits. These include *Ampelocissus martini*, *Antidesma puncticulatum*, *Artocarpus lacucha*, *Calamus viminalis*, *Canthium berberidifolium*, *Dillenia hookeri*, *Embelia subcoriacea*, *Ficus racemosa*, *Flacourtia indica*, *Huberantha cerasoides*, *Lepisanthes rubiginosa*, *Litsea glutinosa*, *Memecylon edule*, *Nephelium hypoleucum*, *Streblus asper*, *Terminalia chebula*, and *Ziziphus oenopolia*.

Some species also provide additional uses beyond fruit consumption. For example, *Calamus viminalis* yields tender shoots eaten as vegetables, *Memecylon edule* provides young shoots in addition to ripe fruits, and *Dillenia hookeri* is cultivated for shade and ornamental purposes, with durable wood used in construction and furniture.

Notably, *Ampelocissus martini* has a culturally specific preparation method; its ripe fruits are sometimes pounded with unripe banana to reduce irritation before consumption, illustrating the Tai Yoy’s traditional knowledge in processing wild fruits.

#### 3.2.3. WEPs Used as Staple Food

Two species of WEPs were recorded as staple foods in AAD (Table 2). Both species are tuberous plants traditionally processed and consumed as carbohydrate-rich staples. *Dioscorea hispida* is prepared by steaming and consumed directly as a staple food or further processed into traditional sweets. *Tacca leontopetaloides* tubers are processed into starch powder through peeling, slicing, grinding, filtering, and drying; the resulting starch is used in various food preparations. These species reflect the Tai Yoy community’s knowledge of processing wild tuberous plants to provide essential dietary carbohydrates, illustrating the integration of WEPs into traditional food systems.

#### 3.2.4. WEPs Used as Sweets/Desserts/Snacks

Three species of WEPs were recorded as sources of sweets, desserts, or snacks in AAD (Table 2). *Canarium subulatum* produces fruits whose seeds are halved and eaten directly as snacks using a small stick. *Momordica cochinchinensis* fruits are incorporated into traditional desserts, while its leaves and shoots are boiled or steamed and consumed as vegetables with chili paste. *Dioscorea hispida* tubers, in addition to being consumed as a staple food, are also processed into traditional sweets after steaming. These species illustrate the versatile use of WEPs by the Tai Yoy, providing not only nutrition but also traditional snack and dessert options within the community.

#### 3.2.5. WEPs Used as Vegetables

In total, 24 WEP species were recorded as being used as vegetables by the Tai Yoy ethnic group in AAD (Table 2). The edible parts include young shoots, leaves, inflorescences, and fruits, which are prepared in different ways according to local culinary traditions. Most are eaten boiled, steamed, or raw with chili paste, depending on taste and texture.

Young shoots are the most frequently used parts. Species such as *Bambusa bambos*, *Gnetum gnemon*, *Hellenia speciosa*, and *Smilax perfoliata* produce tender shoots that are commonly eaten fresh or cooked in local dishes. Leaves of *Careya arborea*, *Paederia linearis*, and *Phyllanthus androgynus* are consumed raw as side vegetables or cooked as ingredients in soups and curries.

Several plants require special preparation to reduce bitterness or acridity. The shoots of *Amorphophallus paeoniifolius* are boiled before cooking, while the leaves of *Senna timoriensis* are blanched to lessen their bitter taste. The inflorescences of *Curcuma angustifolia*, *Markhamia stipulata*, and *Oroxylum indicum* are boiled or eaten fresh with chili paste, a common practice in the region.

Some species have multiple uses within the community. *Calamus viminalis* provides edible fruits and young shoots; *Memecylon edule* offers both fruits and shoots; and *Momordica cochinchinensis* is used not only for its young leaves and shoots as vegetables but also for its fruits in traditional desserts. In addition, *Garcinia cowa* has edible leaves and fruits, while its bark and latex are used as a natural dye for fabrics.

#### 3.2.6. WEPs Used as Medicinal Edible Plants

A total of 35 WEP species were documented as being utilized by the Tai Yoy ethnic group in AAD as both food and traditional herbal ingredients (Table 2). These species are commonly consumed in various forms—fresh, cooked, or as components of local recipes—demonstrating the integration of edible plants into daily life and cultural practices.

Several species showed overlapping uses between medicinal and other food-related categories. For example, *Garcinia cowa* and *Hellenia speciosa* were consumed both as vegetables and medicinal foods, while *Senegalia rugata* served as a condiment as well as a medicinal plant. In addition, *Huberantha cerasoides* and *Terminalia chebula* were utilized as fruits and also recognized as edible plants with medicinal roles.

### 3.3. Toxic or Potentially Harmful WEPs

Some WEPs recorded in this study are known by local people to be potentially toxic or to cause irritation if improperly prepared or consumed in excessive amounts (Table 3). These species require specific local knowledge and preparation methods to ensure safe use.

### 3.4. Cultural Food Significance Index (CFSI) of WEPs

A total of 78 WEP species were evaluated for their cultural medicinal significance using the Cultural Food Significance Index (CFSI) (Table S2). The CFSI values exhibited a broad range, indicating differences in availability, frequency of utilization, diversity of parts used, multifunctionality, taste appreciation, and perceived food–medicinal roles within the local community.

The species with the highest CFSI value was *Hellenia speciosa* (426.08), reflecting its outstanding cultural importance as both a dietary and medicinal resource. This high value was attributed to consistently strong scores across all contributing indices, including high availability (AI = 4), frequency of utilization (FUI = 3), diversity of parts used (PUI = 4.75), multifunctional use (MFFI = 1), taste score appreciation (TSAI = 6.5), and a strong food–medicinal role (FMRI = 5). These results highlight their dual significance in food and traditional healthcare practices.

*Senegalia rugata* (379.08), *Urceola polymorpha* (335.34), *Momordica cochinchinensis* (309.83), and *Spondias pinnata* (255.15) followed as species with high cultural values. These taxa exhibited high utilization frequency and broad recognition in both culinary and medicinal contexts, supported by strong medicinal relevance and favorable taste scores. Their multifunctional uses suggest that these species play essential roles in the maintenance of local food security and folk medicine traditions.

Species with moderate CFSI values, such as *Careya arborea* (178.20), *Tacca leontopetaloides* (170.10), and *Oroxylum indicum* (150.05), are frequently used and hold notable medicinal importance but to a lesser extent than the top-ranked species. These plants are still valued in local traditions, often incorporated into both food and home remedies.

In contrast, species such as *Casearia grewiifolia* (6.60), *Erythroxylum cuneatum* (6.40), and *Litsea glutinosa* (1.86) recorded the lowest CFSI values, indicating limited or specialized ethnomedicinal roles. Their restricted use may be due to ecological scarcity, specific cultural contexts, or specialized therapeutic applications known only among certain informants.

**Figure 5:**
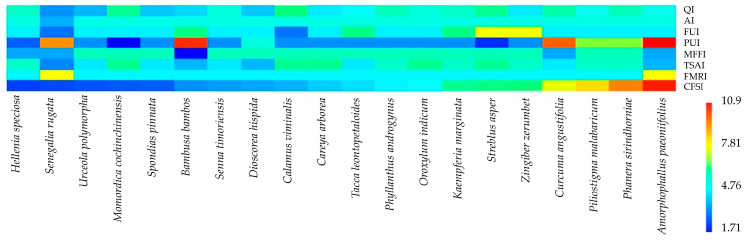
Heat map showing the top 20 wild edible plant species ranked by Cultural Food Significance Index (CFSI) values used by the Tai Yoy ethnic group.

The heat map analysis (Figure 5) illustrates the variation in Cultural Food Significance Index (CFSI) values among the top 20 wild edible plant species used by the Tai Yoy community. The CFSI values ranged from 2.49 to 10.47, indicating marked differences in cultural food importance among species. *Hellenia speciosa* showed the highest CFSI value (10.47), followed by *Senegalia rugata* (9.31), *Urceola polymorpha* (8.24), and *Momordica cochinchinensis* (7.61). These species formed a distinct cluster characterized by high QI, AI, PUI, and FMRI value, reflecting their frequent use, wide availability, and multifunctional roles in the local diet.

Species such as *Bambusa bambos*, *Senna timoriensis*, and *Spondias pinnata* occupied intermediate positions, indicating moderate levels of use and cultural importance. In contrast, *Amorphophallus paeoniifolius* and *Phanera sirindhorniae* had the lowest CFSI values (2.59 and 2.49, respectively), reflecting their limited use or more specific contexts of consumption.

Interestingly, plants with high %PUI and %MFFI values, such as *Phanera sirindhorniae* and *Streblus asper*, exhibited notable versatility in plant parts used and preparation forms, which may contribute to their moderate CFSI rankings despite lower overall frequency of use. Species with low %MFRI values, including *Curcuma angustifolia* and *Zingiber zerumbet*, tended to have weak associations with medicinal uses, explaining their relatively reduced CFSI scores even though they are commonly known edible species.

The integration of component indices within the CFSI framework provides a clear visualization of culturally significant wild edible plants. The observed clustering patterns reveal how factors such as use frequency, availability, and multifunctionality jointly influence the cultural food value of each species within the Tai Yoy community.

### 3.5. Ethnomedicinal of WEPs

#### 3.5.1. Condition of Plants Used and Routes of Administration

**Figure 6:**
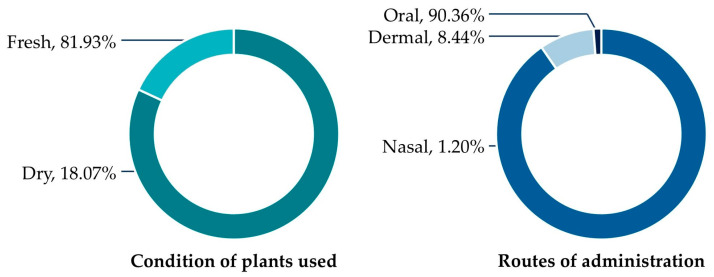
Proportion of condition of plant used and routes of administration.

The condition of plant materials used in the preparation of WEPs reflects the local preferences and practical strategies of resource utilization among the Tai Yoy community (Figure 6, Table S1). Most species (81.93%) were reported to be used in fresh form, indicating a strong reliance on readily available materials collected directly from nearby forests, home gardens, or fallow lands. This practice helps preserve the plants’ natural flavors, nutritional qualities, and perceived therapeutic potency. In contrast, only 18.07% of the cited species were utilized in dried form, mainly for long-term storage, ease of preparation, or incorporation into decoctions. Drying was particularly common for species with seasonal availability or those involved in traditional medicinal preparations requiring prolonged boiling.

Among the WEPs utilized for medicinal purposes, oral administration (90.36%) was overwhelmingly predominant (Figure 5, Table S1). Most remedies were prepared as decoctions, infusions, or consumed directly as food or beverages, reflecting the integration of medicinal and dietary practices within the community—where plants serve both nutritional and therapeutic roles. Dermal application (8.44%) represented the second most common route, involving the external use of crushed or boiled plant materials applied to the skin to treat wounds, inflammation, or dermatological conditions. A minor proportion (1.20%) of medicinal WEPs were administered nasally, typically through inhalation or exposure to plant volatiles to relieve headaches or nasal congestion.

#### 3.5.2. Fidelity Level (%FL) of WEPs

The Fidelity Level (FL) analysis provides insight into the level of cultural agreement among informants regarding the primary medicinal roles of WEPs used by the Tai Yoy ethnic group in AAD (Table S1). FL quantifies the proportion of informants who consistently cited a species for its principal therapeutic purpose, thereby reflecting the perceived efficacy and cultural importance of that use within the community’s traditional healthcare practices.

High FL values indicate strong cultural consensus and confidence in the healing potential of specific plants. In this study, several species showed FL values exceeding 70%, including *Celastrus paniculatus* (77.50%) for stimulating the nervous system, *Dipterocarpus obtusifolius* (72.50%) and *Suregada multiflora* (72.50%) for promoting oral and skin health, *Pueraria mirifica* (72.50%) as a rejuvenating tonic for vitality, and *Ochna integerrima* (70.00%) as a restorative tonic to strengthen the body. Such high FL values demonstrate that these species hold prominent positions in Tai Yoy ethnomedicine and are regarded as reliable and effective remedies.

Moderately high FL values (60–70%) were observed in species such as *Erythroxylum cuneatum* (67.50%) for treating tendon and muscle pain, *Terminalia chebula* (67.50%) as a laxative for gastrointestinal disorders, *Diospyros ehretioides* (65.00%) and *Cryptolepis buchananii* (65.00%) for fever and bruises, respectively, and *Phanera sirindhorniae* (62.50%) for treating chronic skin eruptions. These species are widely known and frequently cited, underscoring their therapeutic importance and consistent use across the Tai Yoy community.

Several species exhibited moderate FL values (40–60%), indicating multifunctional or versatile roles in traditional healing. Examples include *Biancaea sappan* (55.00%) for blood nourishment, *Cynanchum pulchellum* (57.50%) for detoxification and treatment of poisoning, *Curculigo latifolia* (50.00%) for promoting strength, and *Hellenia speciosa* (50.00%) for urinary tract and uterine health. These plants are valued for their broad medicinal utility, though informant consensus is distributed among multiple therapeutic categories.

Lower FL values (<40%) were recorded in species such as *Casearia grewiifolia*, *Connarus semidecandrus*, and *Harrisonia perforata* (each with FLs between 30 and 37.50%), which are employed to treat common ailments like fever, diarrhea, and abdominal discomfort. The lower consensus likely reflects their diverse or secondary medicinal applications, variation in local use practices, or knowledge differences among informants.

#### 3.5.3. Informant Consensus Factor (F_ic_) of WEPS

The Informant Consensus Factor (F_ic_) was calculated to evaluate the level of agreement among informants regarding the use of wild edible plants (WEPs) for treating different therapeutic categories (Table 4). The F_ic_ values in this study ranged from 0.944 to 1.000, indicating a generally high degree of shared ethnomedicinal knowledge within the Tai Yoy community.

The highest F_ic_ value (1.000) was observed for the poisoning/toxicology category, suggesting complete consensus among informants on the use of a single plant species for this condition. Similarly, high F_ic_ values were found for central nervous system disorders (0.982), musculoskeletal disorders (0.968), and eye disorders (0.968), reflecting strong agreement on the specific plants used to alleviate these ailments.

The gastrointestinal disorders category exhibited the largest number of use reports (N_ur_ = 379) and species (N_t_ = 18), with a F_ic_ value of 0.955, highlighting its importance and diversity in the local health care system. Other categories, including reproductive disorders (0.952), skin disorders (0.950), obstetrics, gynaecology and urinary disorders (0.948), infection/immune disorders (0.945), and blood disorders (0.944), also demonstrated high levels of informant agreement.

### 3.6. Comparative of WEPs from AAD and Surrounding Regions

The Jaccard’s similarity index (JI) was employed to assess the floristic similarity of WEPs documented in AAD with those reported from nearby regions in northeastern Thailand (Table 5). The analysis revealed that AAD shared the highest similarity with Don Pu Ta Forest, Pla Lo Sub-district, Waritchaphum District, Sakon Nakhon Province (JI = 0.094; SM = 9.40%), followed closely by Don Pu Ta Forest, Kut Bak District, Sakon Nakhon Province (JI = 0.084; SM = 8.40%). This indicates that these areas, located within the same province, possess comparable ecological conditions and plant resource utilization patterns.

Moderate similarity was observed between AAD and Khong Chai District, Kalasin Province (JI = 0.076; SM = 7.60%), suggesting overlapping ethnobotanical knowledge and shared species due to geographical proximity and cultural exchanges. In contrast, Ban Phue District, Udon Thani Province (JI = 0.039; SM = 3.90%) and Muang District, Kalasin Province (JI = 0.019; SM = 1.90%) exhibited lower similarity values, reflecting variations in local flora and ethnobotanical traditions. The lowest similarity was found with Huai Mek District, Kalasin Province (JI = 0.008; SM = 0.80%), possibly due to differences in habitat types, vegetation structures, and cultural practices regarding plant use.

**Figure 7:**
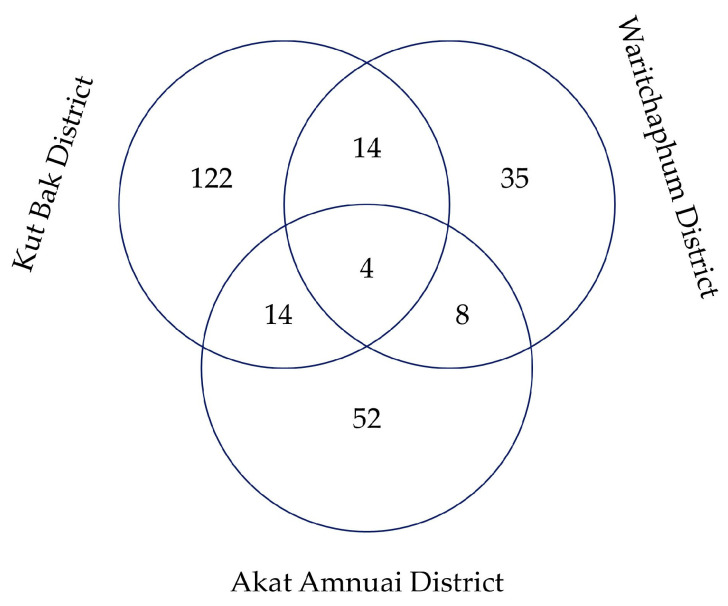
Venn diagram showing the shared and unique wild edible plant species among Akat Amnuai, Waritchaphum, and Kut Bak Districts in Sakon Nakhon Province, Thailand.

A comparison of WEPs among Akat Amnuai, Waritchaphum [10], and Kut Bak Districts [32] highlights similar patterns. The Venn diagram indicates that four species are shared across all three locations (Figure 7), whereas 52 species are newly recorded for Sakon Nakhon Province, having not been reported in previous studies from Waritchaphum and Kut Bak Districts.

### 3.7. Economic Value of WEPs

**Figure 8:**
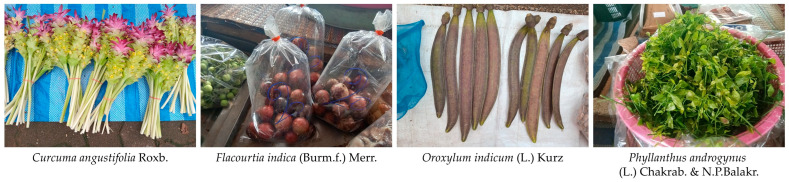
Representative wild edible plant species observed being traded in local markets (photos by Tammanoon Jitpromma).

The trade of WEPs in local markets contributes significantly to household income and local livelihoods. A total of 25 WEP species were recorded as being sold (Table 6, Figure 8), representing a wide range of plant parts including fruits, leaves, tubers, inflorescences, rhizomes, and young shoots. Prices varied considerably among species, ranging from 10 to 160 THB/kg, depending on the plant part, seasonal availability, and consumer demand.

Among the recorded species, *Phyllanthus androgynus* (leaf) showed the highest market value, with an average price of 140–160 THB/kg and generating the greatest average yearly income per trader (10,170 THB). Other economically important species included *Curcuma angustifolia* (inflorescence; 4401 THB/year), *Spondias pinnata* (fruit; 4260 THB/year), and *Tacca leontopetaloides* (tuber; 3675 THB/year). In contrast, species such as *Ficus racemosa* and *Terminalia chebula* provided lower returns, with yearly incomes below 600 THB per trader.

Seasonal availability also influenced market dynamics, with most species available for only 3–4 months annually. The highest monthly sales volumes were observed for *Phyllanthus androgynus* (22.6 kg), *Bambusa bambos* (21.3 kg), and *Antidesma puncticulatum* (20.1 kg). Species with broader seasonal availability (4–6 months) generally contributed more consistently to traders’ income.

## 4. Discussion

### 4.1. Diversity of WEPs and Implications for Food Sustainability and Conservation

A total of 78 wild edible plant species, belonging to 74 genera and 42 families, were recorded among the Tai Yoy people in AAD. This richness reflects both the floristic diversity of the area and the community’s deep understanding of their surrounding environment. The predominance of Fabaceae, Apocynaceae, and Zingiberaceae aligns with other ethnobotanical studies in Thailand [36,37]. These families are ecologically abundant and functionally important due to their adaptive traits and rich secondary metabolites. Fabaceae thrive in poor soils through nitrogen fixation and provide protein-rich edible parts [38]. Apocynaceae species contain pharmacologically active compounds and tolerate dry or disturbed habitats, increasing accessibility [39]. Zingiberaceae are widespread in moist forests and homegardens, with aromatic rhizomes used in both food and medicine [40]. These ecological adaptability traits and nutritional and therapeutic properties explain their consistent cultural importance across multiple regions.

The majority of species (almost all) were native to Thailand, suggesting that the Tai Yoy people continue to depend largely on indigenous flora. Such reliance on native resources contributes directly to food sustainability, as local species are well adapted to the environment and require minimal external inputs [41]. The predominance of trees and climbers suggests that the Tai Yoy people utilize species that are relatively abundant and accessible in their surroundings, offering reliable seasonal resources for food and medicine [42]. Future studies could incorporate ecological surveys to analyze species-habitat associations, seasonal availability, and population dynamics, which would provide deeper insights into sustainable harvesting practices and resource management.

Fruits and leaves were the most frequently used parts, indicating a preference for renewable resources that support sustainable harvesting. Similar patterns have been observed in studies from Yasothon Province, Thailand [22], and Sarawak, Malaysia [43], suggesting that communities across Southeast Asia share comparable traditions of using accessible and regenerable plant parts in their diets. This resemblance reflects a broader cultural convergence in the way local groups integrate wild plants into daily food practices, emphasizing both sustainability and the transmission of traditional ecological knowledge. Although roots and whole plants were used only occasionally, such practices highlight the importance of monitoring harvesting impacts and considering conservation awareness, particularly for species that may be vulnerable due to their growth characteristics [44].

Approximately one-third of the recorded plants are traded in local markets, linking traditional knowledge to rural livelihoods. Promoting sustainable harvest and cultivation practices for these species will help maintain biodiversity, strengthen local food systems, and align with Thailand’s Bio-Circular-Green (BCG) economy goals [45].

### 4.2. Utilization Patterns of WEPs: Food, Medicine, and Culinary Practices

Several WEPs, including *Piliostigma malabaricum*, *Senegalia rugata*, and *Spondias pinnata*, are widely used as natural flavor enhancers. They enrich the taste and aroma of traditional dishes, reflecting a Southeast Asian culinary pattern in which sour, bitter, and aromatic plants play key roles. *Piliostigma malabaricum* has been reported in Nigeria as being cooked with fish or meat to enhance soup flavors, a practice similar to traditional preparation methods in northeastern Thailand [46]. *Senegalia rugata* is also used as a seasoning, particularly in northern Thailand, demonstrating the widespread and culturally specific use of these plants [47]. *Spondias pinnata* is commonly added to “som tam” (papaya salad), representing a shared food culture between Thailand and Laos [48]. These examples highlight both regional variation and the common principle of utilizing wild plants to improve flavor while maintaining traditional food practices.

Eighteen species were consumed primarily as fruits, providing important seasonal nutrition. Most fruits were eaten fresh, while some, such as *Ampelocissus martini*, required specific preparation to reduce irritation or toxicity. Starchy tubers, including *Dioscorea hispida* and *Tacca leontopetaloides*, served as staple foods or fallback resources during periods of scarcity. These practices reflect the Tai Yoy community’s detailed traditional knowledge in identifying, processing, and safely consuming wild plants, demonstrating how ecological understanding is integrated into daily dietary practices [49].

Vegetables represented the largest category, with 24 species providing edible shoots, leaves, and inflorescences, often collected from forests or fallow lands. Many species also serve medicinal functions, including *Garcinia cowa* and *Hellenia speciosa*, illustrating the integration of food and medicine in daily practice. While most WEPs are safe for consumption, certain species require careful processing to avoid toxicity, such as *Dioscorea hispida* and *Gluta usitata*. Knowledge of safe preparation methods highlights the community’s expertise in managing wild resources responsibly, ensuring both nutrition and health protection [50,51].

The multifunctional use of WEPs supports dietary diversity, strengthens food security, and contributes to the sustainable use of local biodiversity. By maintaining traditional gathering practices and ecological knowledge, the community preserves cultural heritage while promoting long-term conservation of wild plant resources.

It should be acknowledged that the use categories applied in this study—vegetables, fruits, condiments, staple foods, sweets, and medicinal edible plants—are not entirely homogeneous. These categories combine botanical, culinary, nutritional, and functional criteria, and in many cases are not mutually exclusive. Such overlap reflects the complexity of Thai food culture, where a single plant species may be consumed in multiple forms, prepared in diverse ways, or used simultaneously as food, medicine, and flavoring. While this classification helps structure the analysis, it may introduce minor biases that could overestimate or underestimate the cultural value of certain multipurpose species.

This integration of food and medicinal use reflects the daily practices and local knowledge of the Tai Yoy people. It underscores the cultural and ecological significance of these species and highlights the importance of preserving traditional knowledge alongside biodiversity.

### 4.3. Cultural Food Significance and Implications for Sustainability

The Cultural Food Significance Index (CFSI) provides a framework to understand how wild edible plants (WEPs) are valued within the Tai Yoy community for both dietary and medicinal purposes. Species with the highest CFSI values, such as *Hellenia speciosa*, *Momordica cochinchinensis*, *Senegalia rugata*, and *Urceola polymorpha*, highlight the central role of multifunctional plants in daily life. Their high scores reflect widespread availability, frequent use, diverse plant parts, favorable taste, and integration into medicinal practices. These findings emphasize the close connection between food and medicine in Tai Yoy traditions. Given their cultural and functional importance, these species should be prioritized in conservation initiatives, including sustainable harvesting and habitat protection, to ensure continued access to essential food and medicinal resources [52,53].

Species with moderate CFSI values, such as *Careya arborea* and *Tacca leontopetaloides*, continue to contribute to household nutrition and health, although their use is less frequent or regionally specific. In contrast, species with low CFSI values, including *Casearia grewiifolia* and *Litsea glutinosa*, reflect either limited ecological distribution or specialized uses known only to a few informants. These findings highlight that cultural importance is closely linked to both ecological factors and knowledge transmission within the community, emphasizing the need to preserve traditional practices alongside the plants themselves [54,55].

The heat map analysis of CFSI values reinforces the concept of multifunctionality as a key determinant of cultural importance. Species that are frequently used, widely available, and versatile in their preparation occupy central positions in the community’s food and medicinal systems, whereas species with restricted use, limited medicinal relevance, or specialized culinary applications rank lower [56]. Notably, even species with lower overall CFSI values may exhibit high versatility in plant parts used or preparation methods, suggesting nuanced local knowledge that allows communities to optimize the use of available resources [57].

From a sustainability perspective, the integration of high-CFSI species into daily diets and traditional medicine promotes resilient food systems. These species provide seasonal nutrition, medicinal support, and cultural continuity, while their multifunctional use reduces the need to rely on external food sources [58]. Conservation of these culturally significant WEPs is therefore essential, particularly for species that are harvested extensively or have slow growth rates. Maintaining traditional ecological knowledge, including sustainable harvesting and processing methods, ensures both the preservation of biodiversity and the long-term resilience of local food systems [59].

Overall, the CFSI framework offers a comprehensive understanding of how cultural, ecological, and medicinal factors shape the use of WEPs. The findings underscore the interconnection between food and medicine in Tai Yoy traditions, highlight species of high cultural value, and provide evidence for the need to integrate ethnobotanical knowledge into strategies for food sustainability and conservation in Northeastern Thailand.

It should be noted that while the current analysis focuses on dietary and medicinal uses, several wild edible plants documented in this study also serve additional purposes, including construction, ornamentation, ritual, and other utilitarian functions. These additional uses were not incorporated into the CFSI evaluation, which may lead to an underestimation of the broader cultural significance of certain multipurpose species. Future studies incorporating all functional uses would provide a more comprehensive assessment of the cultural and ecological value of these plants within the Tai Yoy community.

### 4.4. Medicinal Significance of WEPs

The ethnomedicinal practices of the Tai Yoy community reflect a deep integration of wild edible plants (WEPs) within the food–medicine continuum. The predominant use of fresh plant materials highlights reliance on readily available species that retain both nutritional and therapeutic properties. Occasional use of dried materials indicates adaptive strategies for coping with seasonal scarcity and storage needs, reflecting empirical knowledge on how processing affects efficacy and bioactive compound stability [60].

Oral administration is the dominant mode of use, consistent with dietary integration, while dermal and nasal applications are employed more selectively for conditions such as wounds, inflammation, and headaches. This pattern underscores how the Tai Yoy community embeds wild-plant knowledge across both nutritional and therapeutic domains, demonstrating a flexible and context-sensitive approach to health management [61,62].

Fidelity level (FL) and Informant Consensus Factor (F_ic_) analyses reveal which species are culturally and therapeutically significant. High consensus for certain plants indicates strong agreement on efficacy for specific ailments, whereas moderate or low values suggest multifunctional use distributed across various conditions. These trends emphasize adaptive knowledge, where plants serve multiple roles depending on seasonal availability, ecological context, and local health needs [63,64,65].

Overall, the findings highlight that WEPs are not merely supplemental foods but integral components of health and well-being. The multifunctionality of high-value species supports dietary diversity, traditional medicine, and cultural continuity. Recognizing this dual significance informs conservation priorities: protecting habitats, ensuring sustainable harvesting, and preserving local ecological knowledge are essential to maintain both biodiversity and resilient food–medicine systems for the Tai Yoy community.

### 4.5. Regional Similarity and Ethnobotanical Diversity of WEPs

The comparative analysis using Jaccard’s similarity index (JI) revealed that the WEPs diversity in AAD is most similar to nearby forest areas in Sakon Nakhon Province, particularly Don Pu Ta Forest in Pla Lo and Kut Bak Subdistricts. This suggests that these regions share comparable ecological conditions and resource-use patterns, likely influenced by similar climate and forest types [66]. Moderate similarity with Khong Chai District in Kalasin Province further indicates the role of geographic proximity and historical cultural exchanges among local ethnic communities [67]. In contrast, relatively low similarity values with Ban Phue, Muang, and Huai Mek Districts highlight the impact of ecological heterogeneity, diverse vegetation types, and distinct cultural traditions on plant use. These patterns underscore how local environmental settings and sociocultural histories shape ethnobotanical knowledge and species utilization [68].

Overall, the observed patterns reflect both ecological continuity and cultural specificity within northeastern Thailand. Regions with shared landscapes and ethnic ties tend to maintain overlapping ethnobotanical repertoires, whereas ecologically distinct or more distant areas show divergence in species composition and plant-use practices. These findings emphasize the need for localized conservation strategies, as ethnobotanical diversity can vary substantially even within the same province, reflecting unique community adaptations and ecological relationships.

Comparisons with WEPs documented in Sakon Nakhon Province reveal both overlap and distinctiveness in plant use. Notably, 52 newly recorded species in this study appear unique to the Tai Yoy community, reflecting localized ecological knowledge and culturally specific practices. These species appear to reflect unique aspects of the Tai Yoy community’s ecological knowledge, culinary preferences, and medicinal practices. Sociocultural factors—such as traditional food preparation methods, local seasonal availability, spiritual beliefs, and community-specific knowledge transmission—likely shape the distinct use patterns observed in this group, highlighting both cultural specificity and adaptation to local environmental conditions. The presence of numerous unrecorded species reflects the community’s close ecological adaptation and reliance on surrounding forest and fallow land resources [69,70]. It also demonstrates that data from a single district cannot capture the full spectrum of plant use across the province, reinforcing the importance of localized surveys for comprehensive biodiversity documentation [71,72].

Moreover, these newly recorded species represent potential resources for nutritional and pharmacological studies, as well as priorities for local conservation efforts [73]. Protecting these species and sustaining traditional knowledge are crucial for maintaining both cultural identity and ecological resilience in northeastern Thailand [74].

### 4.6. Economic Value of WEPs and Implications for Sustainable Use

The trade of wild edible plants (WEPs) in local markets provides an important source of income for households, with 25 species sold, covering diverse plant parts such as fruits, leaves, tubers, inflorescences, rhizomes, and young shoots. The most economically valuable species—*Curcuma angustifolia*, *Phyllanthus androgynus*, *Spondias pinnata*, and *Tacca leontopetaloides*—also correspond to those with high Cultural Food Significance Index (CFSI) values, suggesting an overlap between cultural importance and market demand. These species are widely recognized for both food and medicinal uses, and their multifunctionality likely contributes to their prominent role in local diets and traditional healthcare practices.

Seasonal availability strongly influences market dynamics. Species available only 3–4 months per year may experience concentrated harvesting pressure, whereas those with extended availability tend to contribute more consistently to traders’ income. The highest monthly sales volumes, observed for *Phyllanthus androgynus*, *Bambusa bambos*, and *Antidesma puncticulatum*, illustrate how availability, consumer preference, and plant part versatility interact to determine economic significance.

The combination of high cultural and economic value could raise conservation concerns. Many high-demand species are primarily collected from wild habitats, and although current use appears sustainable, unregulated or intensive harvesting could potentially threaten local populations over time [75]. Promoting the cultivation of high-CFSI and high-market-value species in home gardens or community plots may help reduce pressure on wild stocks while providing better control over quality, harvest timing, and year-round supply, thereby supporting local livelihoods [76].

Integrating WEPs into agroforestry and mixed cropping systems can further contribute to food security, support the maintenance of traditional ecological knowledge, and help preserve local biodiversity [77]. Such approaches may assist in ensuring that culturally and economically important species continue to benefit the Tai Yoy community while minimizing impacts on natural populations.

### 4.7. Novelty of the Study

This study documents the ethnobotany of WEPs among the Tai Yoy community in AAD, Sakon Nakhon Province, and highlights several new findings. A total of 78 WEP species were recorded, of which 52 species are reported for the first time in Sakon Nakhon Province. These newly recorded species demonstrate the community’s extensive traditional knowledge and the high diversity of locally available wild plants. The results also show how the Tai Yoy people are closely adapted to their surrounding forests and fallow lands, relying on these habitats for both food and medicinal resources.

The study combines field surveys with quantitative analyses, including Jaccard’s similarity index, Cultural Food Significance Index (CFSI), fidelity level (FL), and market-based economic evaluation. This approach provides a detailed understanding of WEPs from ecological, cultural, medicinal, and economic perspectives. By documenting the multifunctional use of these plants in daily diets, traditional medicine, and local markets, the research emphasizes the importance of culturally significant species for both community livelihoods and biodiversity conservation.

Overall, the study offers a region-specific and comprehensive dataset that fills gaps in current ethnobotanical knowledge for northeastern Thailand and provides a foundation for future research, conservation planning, and sustainable management of WEP resources.

### 4.8. Suggestions for Future Research

Building on the findings and limitations of this study, several directions for future research are recommended. First, expanding surveys to additional districts and provinces would help capture the full spectrum of WEP diversity and local ethnobotanical knowledge in northeastern Thailand, clarifying whether the high number of newly recorded species in AAD reflects a unique local pattern or broader regional trends. Second, studies on cultivation and domestication are needed to investigate propagation methods, growth performance, and agroecological suitability of high-demand WEPs, supporting sustainable cultivation and reducing pressure on wild populations. Third, nutritional and pharmacological analyses of both newly recorded and culturally significant species would provide insight into their dietary and medicinal value, ensuring safe and evidence-based utilization. Fourth, ethnoecological and conservation research could examine the relationships between harvesting practices, seasonal availability, and ecological sustainability, informing targeted conservation strategies and integrating traditional knowledge into biodiversity management. Finally, market and livelihood studies focusing on supply chains, consumer preferences, and the economic impacts of WEP trade would identify opportunities for sustainable commercialization and equitable benefit-sharing within local communities. Collectively, these approaches would strengthen the connections between ethnobotanical knowledge, biodiversity conservation, and sustainable development, ensuring that WEP resources continue to support both cultural heritage and ecological resilience in northeastern Thailand.

## 5. Conclusions

This study records a rich diversity of 78 wild edible plant (WEP) species from 42 families among the Tai Yoy community in Akat Amnuai District, Sakon Nakhon Province, reflecting a deep ethnobotanical heritage and close ecological knowledge. The predominance of native species, alongside plants harvested from forests, fallow lands, and home gardens, highlights adaptive strategies that support local food security, nutrition, and cultural identity.

Quantitative analyses, including Cultural Food Significance Index (CFSI), fidelity level (FL), and market valuation, reveal the multifunctional roles of WEPs. Species such as *Curcuma angustifolia*, *Phyllanthus androgynus*, and *Spondias pinnata* exemplify the integration of food, medicine, and income generation within local livelihoods. High FL and consensus values demonstrate strong cultural agreement on the use and therapeutic benefits of key species, reflecting the close link between traditional knowledge and health practices.

The diversity of plant uses—from fruits and leaves to tubers, shoots, and inflorescences—illustrates a holistic approach to food systems that balances multifunctionality and specialization. This complexity enhances dietary diversity, supports resilience, and provides critical insights for biodiversity conservation and sustainable development. Promoting cultivation of high-demand species could reduce pressure on wild populations while maintaining access to culturally significant plants.

Ultimately, this ethnobotanical investigation emphasizes the importance of conserving both plant biodiversity and indigenous knowledge to strengthen food sovereignty, cultural continuity, and community well-being. Future research could explore the nutritional and pharmacological properties of key species, sustainable cultivation methods, and strategies for knowledge transmission, thereby ensuring the persistence of ethnobotanical heritage and supporting resilient, locally adapted food and health systems.

## Figures and Tables

**Table 1:** Number of species (NoP) and percentage of wild edible plant families recorded from the study area.

Family	NoP	%	Family	NoP	%	Family	NoP	%
Fabaceae	7	8.97	Euphorbiaceae	2	2.56	Costaceae	1	1.28
Apocynaceae	5	6.41	Hypericaceae	2	2.56	Cucurbitaceae	1	1.28
Zingiberaceae	4	5.13	Lauraceae	2	2.56	Dilleniaceae	1	1.28
Anacardiaceae	3	3.85	Phyllanthaceae	2	2.56	Erythroxylaceae	1	1.28
Bignoniaceae	3	3.85	Salicaceae	2	2.56	Gnetaceae	1	1.28
Moraceae	3	3.85	Sapindaceae	2	2.56	Hypoxidaceae	1	1.28
Rubiaceae	3	3.85	Vitaceae	2	2.56	Lecythidaceae	1	1.28
Rutaceae	3	3.85	Amaranthaceae	1	1.28	Melastomataceae	1	1.28
Arecaceae	2	2.56	Annonaceae	1	1.28	Ochnaceae	1	1.28
Combretaceae	2	2.56	Asteraceae	1	1.28	Poaceae	1	1.28
Connaraceae	2	2.56	Burseraceae	1	1.28	Primulaceae	1	1.28
Dioscoreaceae	2	2.56	Capparaceae	1	1.28	Rhamnaceae	1	1.28
Dipterocarpaceae	2	2.56	Celastraceae	1	1.28	Smilacaceae	1	1.28
Ebenaceae	2	2.56	Clusiaceae	1	1.28	Stemonaceae	1	1.28

**Table 2:** Inventory of wild edible and multipurpose plant species recorded in the study area, including family, scientific name, vernacular name (VE), distribution in Thailand (DiT), growth habit (GH), utilization, used parts, methods of utilization, trade in local market (TiM), and voucher number (VN).

No.	Family	Scientific Name	VE	DiT	GH	Utilization	Used Parts	Method of Utilization	TiM	VN
1.	Amaranthaceae	*Achyranthes aspera* L.	Ya Phan Ngu	Native	Herb	MP	Lv, St, Wp	Medicinal uses detailed in Table S1	No	TJ589
2.	Anacardiaceae	*Gluta usitata* (Will.) Ding Hou	Nam Kliang	Native	Tree	MP	Bk, La, St	Latex: Used to produce natural varnish. The clear latex turns black and glossy upon exposure to air and is applied as a priming lacquer on wooden surfaces in the traditional “Long Rak–Pid Thong” (lacquer and gold gilding) process for making lacquerware; Medicinal uses detailed in Table S1	No	TJ628
3.	Anacardiaceae	*Mangifera caloneura* Kurz	Ma Muang Ka So	Native	Tree	FT	Ft	Fruit: The fruits are edible both raw and ripe and commonly consumed as fruits	No	TJ637
4.	Anacardiaceae	*Spondias pinnata* (L.f.) Kurz	Ma Kok	Native	Tree	CF	Ft	Fruit: The sour–sweet fruits are used as a flavoring ingredient in various dishes to enhance taste	Yes	TJ655
5.	Annonaceae	*Huberantha cerasoides* (Roxb.) Chaowasku	Sai Den	Native	Tree	MP, FT	Ft, Lv, Rt	Fruit: The ripe fruits are eaten fresh as seasonal fruits; Medicinal uses detailed in Table S1	No	TJ632
6.	Apocynaceae	*Amphineurion marginatum* (Roxb.) D.J.Middleton	Khreu Sai Tan	Native	Climber	MP	Rt, St	Medicinal uses detailed in Table S1	No	TJ592
7.	Apocynaceae	*Cryptolepis buchananii* R.Br. ex Roem. & Schult.	En On	Native	Climber	MP	Lv, St	Medicinal uses detailed in Table S1	No	TJ611
8.	Apocynaceae	*Cynanchum pulchellum* (Wall.) Liede & Khanum	Ton Khao San	Native	Climber	MP	Rt	Medicinal uses detailed in Table S1	No	TJ614
9.	Apocynaceae	*Streptocaulon juventas* (Lour.) Merr.	Khreu Pra Song	Native	Climber	MP	Rt	Medicinal uses detailed in Table S1	No	TJ658
10.	Apocynaceae	*Urceola polymorpha* (Pierre ex Spire) D.J.Middleton & Livsh.	Som Lom	Native	Climber	CF	Ft, Lv	Fruit, Leaf: Used in cooking to impart a sour flavor	No	TJ664
11.	Araceae	*Amorphophallus paeoniifolius* (Dennst.) Nicolson	I Rok	Native	Herb	VG	St	Shoot: Boiled to remove acridity before being cooked as a vegetable dish	Yes	TJ590
12.	Arecaceae	*Calamus viminalis* Willd.	Wai	Native	Climber	FT	Ft, St	Fruit: The ripe fruits are eaten as fruits; Shoot: young shoots are used as a vegetable ingredient	Yes	TJ598
13.	Asteraceae	*Elephantopus scaber* L.	King Fai Nok Khum	Native	Herb	MP	Lv, Rt	Medicinal uses detailed in Table S1	No	TJ621
14.	Bignoniaceae	*Fernandoa adenophylla* (Wall. ex G.Don) Steenis	Kha Rao	Native	Tree	VG	Ic	Inflorescence: Boiled and eaten as a blanched vegetable with chili paste or cooked as part of various dishes	No	TJ624
15.	Bignoniaceae	*Markhamia stipulata* (Wall.) Seem.	Khae Hua Mu	Native	Tree	VG	Ic	Inflorescence: Boiled and eaten as a blanched vegetable with chili paste or added to curries	No	TJ638
16.	Bignoniaceae	*Oroxylum indicum* (L.) Kurz	Lin Fa	Native	Tree	VG	Ft, Ic	Fruit: Fresh or roasted fruits are eaten with chili paste or used in local dishes; Inflorescence: Eaten raw or boiled as a side vegetable with chili paste or cooked in traditional recipes	Yes	TJ644
17.	Burseraceae	*Canarium subulatum* Guillaumin	Bak Liam	Native	Tree	SW	Ft	Fruit: The fruit is halved, and the seeds inside are eaten as a snack using a small stick	No	TJ599
18.	Capparaceae	*Capparis flavicans* Kurz	Ngua Lia	Native	Shrub	MP	Hw, Lv, Wp	Whole Plant: Cultivated as an ornamental plant; Medicinal uses detailed in Table S1	No	TJ601
19.	Celastraceae	*Celastrus paniculatus* Willd.	Khreu Mak Taek	Native	Climber	MP	Hw, Lv	Medicinal uses detailed in Table S1	No	TJ606
20.	Clusiaceae	*Garcinia cowa* Roxb. Ex Choisy	Mak Mong	Native	Tree	MP, VG	Bk, Ft, La, Lv, Rt	Bark, Latex: The yellow bark and latex are used for dyeing fabrics; Fruit, Leaf: The sour parts are used as flavoring ingredients in curries or eaten fresh as vegetables; Medicinal uses detailed in Table S1	No	TJ627
21.	Combretaceae	*Terminalia chebula* Retz.	Som Mo	Native	Tree	MP, FT, VG	Ft	Fruit: The astringent-sour fruits are eaten fresh or processed into pickles; Medicinal uses detailed in Table S1	Yes	TJ662
22.	Combretaceae	*Terminalia elliptica* Willd.	Chuak	Native	Tree	MP	Bk, Hw	Bark provides tannins for leather tanning; Wood: The hardwood is highly durable and polished, used in flooring, beams, furniture, and tool handles, especially for tools used in water; Medicinal uses detailed in Table S1	No	TJ663
23.	Connaraceae	*Connarus semidecandrus* Jack	Khreu Thop Thaeb	Native	Climber	MP	Bk, Rt, Wp	Medicinal uses detailed in Table S1	No	TJ608
24.	Connaraceae	*Rourea stenopetala* (Griff.) Hook.f.	Ma Kham Khreu	Native	Climber	MP	Ft	Medicinal uses detailed in Table S1	No	TJ651
25.	Costaceae	*Hellenia speciosa* (J.Koenig) S.R.Dutta	Ueang Mai Na	Native	Herb	MP, VG	St, Wp, Rz	Shoot: Young shoots are used as a vegetable; Whole Plant: Grown as an ornamental species; Medicinal uses detailed in Table S1	Yes	TJ631
26.	Cucurbitaceae	*Momordica cochinchinensis* (Lour.) Spreng.	Fak Khao	Native	Climber	SW, VG	Ft, Lv, St	Fruit: Used as an ingredient in traditional desserts; Leaf, Shoot: Boiled or steamed and eaten as vegetables with chili paste	Yes	TJ641
27.	Dilleniaceae	*Dillenia hookeri* Pierre	San Din	Native	Herb	FT	Wp	Whole Plant: Cultivated for shade and garden decoration; the durable wood is used in house construction and furniture making	No	TJ615
28.	Dioscoreaceae	*Dioscorea hispida* Dennst.	Kloy	Native	Herb	SF, SW	Tb	Tuber: Steamed and eaten as a staple food or made into traditional sweets	Yes	TJ616
29.	Dioscoreaceae	*Tacca leontopetaloides* (L.) Kuntze	Thao Yai Mom	Native	Herb	SF	Tb	Tuber: Processed into starch powder by peeling, slicing, grinding, filtering through cloth, and drying the sediment; the starch is used in food preparation	Yes	TJ661
30.	Dipterocarpaceae	*Dipterocarpus obtusifolius* Teijsm. ex Miq.	Chad	Native	Tree	MP	Bk, Lv, St	Bark: Used as a natural dye yielding a dull yellow color; Shoot: Used as building material such as posts or firewood; Medicinal uses detailed in Table S1	No	TJ619
31.	Dipterocarpaceae	*Dipterocarpus tuberculatus* Roxb.	Kung	Native	Tree	MP	Lv, St	Leaf: Used for thatching huts or roofing; Shoot: Utilized for making furniture and building structures; Medicinal uses detailed in Table S1	No	TJ620
32.	Ebenaceae	*Diospyros ehretioides* Wall. ex G.Don	Tab Tao Ton	Native	Tree	MP	Rt	Medicinal uses detailed in Table S1	No	TJ617
33.	Ebenaceae	*Diospyros mollis* Griff.	Khuea Ka	Native	Tree	MP	Bk, Ft	Medicinal uses detailed in Table S1	No	TJ618
34.	Erythroxylaceae	*Erythroxylum cuneatum* (Miq.) Kurz	Krai Thong	Native	Shrub	MP	Bk, Rt	Medicinal uses detailed in Table S1	No	TJ623
35.	Euphorbiaceae	*Strophioblachia fimbricalyx* Boerl.	Ba Sad	Native	Shrub	VG	Ic, Lv	Inflorescence, Leaf: Blanched and eaten as vegetables with chili paste	No	TJ659
36.	Euphorbiaceae	*Suregada multiflora* (A.Juss.) Baill.	Duk Sai	Native	Shrub	MP	Bk, Rt	Medicinal uses detailed in Table S1	No	TJ660
37.	Fabaceae	*Biancaea sappan* (L.) Tod.	Sa Fang	Native	Tree	MP	Hw, St	Shoot: Used to produce red dye and for tanning animal hides; Medicinal uses detailed in Table S1	No	TJ596
38.	Fabaceae	*Peltophorum dasyrhachis* (Miq.) Kurz	A Rang	Native	Tree	MP	Bk, Wp	Bark: Used as a natural dye to yield a reddish-brown color; Whole Plant: Grown as an ornamental species; Medicinal uses detailed in Table S1	No	TJ646
39.	Fabaceae	*Phanera sirindhorniae* (K.Larsen & S.S.Larsen) Mackinder & R.Clark	Pra Dong	Native	Tree	MP	St, Wp	Whole Plant: Grown as an ornamental species; Medicinal uses detailed in Table S1	No	TJ647
40.	Fabaceae	*Piliostigma malabaricum* (Roxb.) Benth.	Som Siao	Native	Tree	CF	Lv	Leaf: Used in cooking to impart sour flavor	No	TJ649
41.	Fabaceae	*Pueraria mirifica* Airy Shaw & Suvat.	Khreu Kraw	Native	Climber	MP	Tb	Medicinal uses detailed in Table S1	No	TJ650
42.	Fabaceae	*Senegalia rugata* (Lam.) Britton & Rose	Som Poi	Introduced	Climber	MP, CF	Ft, Lv	Fruit, Leaf: Sour fruits and leaves are used as flavoring in various dishes; Fruit: Mature fruits are sun-dried and boiled with turmeric to prepare a traditional hair-washing solution; Medicinal uses detailed in Table S1	Yes	TJ652
43.	Fabaceae	*Senna timoriensis* (DC.) H.S.Irwin & Barneby	Khi Lek Pa	Native	Tree	VG	Lv	Leaf: Boiled to reduce bitterness before consumption as a vegetable	No	TJ653
44.	Gnetaceae	*Gnetum gnemon* L.	Liang	Native	Climber	VG	Ft	Fruit, Leaf, Shoot: Consumed raw as fresh vegetables or cooked in local dishes	No	TJ629
45.	Hypericaceae	*Cratoxylum cochinchinense* (Lour.) Blume	Tiu Som	Native	Tree	CF	Ic	Inflorescence, Leaf: Used in cooking to enhance sourness or flavor	Yes	TJ609
46.	Hypericaceae	*Cratoxylum formosum* (Jack) Benth. & Hook.f. ex Dyer	Tiu Nam	Native	Tree	CF	Ic	Inflorescence, Leaf: Used in cooking to enhance sourness or flavor	Yes	TJ610
47.	Hypoxidaceae	*Curculigo latifolia* Dryand. ex W.T.Aiton	Wan Sak Lek	Native	Herb	MP	Lv, Rt	Medicinal uses detailed in Table S1	No	TJ612
48.	Lauraceae	*Cassytha filiformis* L.	Sangwan Phra In	Native	Climber	MP	St	Medicinal uses detailed in Table S1	No	TJ604
49.	Lauraceae	*Litsea glutinosa* (Lour.) C.B.Rob.	Mi Men	Native	Tree	FT	Ft	Fruit: Eaten ripe as fruits	No	TJ636
50.	Lecythidaceae	*Careya arborea* Roxb.	Kra Don	Native	Tree	VG	Lv	Leaf: Consumed raw as a fresh vegetable	No	TJ602
51.	Melastomataceae	*Memecylon edule* Roxb.	Muead Ae	Native	Tree	FT, VG	Ft, St, Wp	Fruit: Ripe fruits are eaten; young shoots are eaten as fresh vegetables; Shoot: young shoot eaten as fresh vegetables; Whole Plant: Grown as an ornamental plant	No	TJ639
52.	Moraceae	*Artocarpus lacucha* Buch.-Ham.	Ma Hat	Native	Tree	FT	Ft	Fruit: Ripe fruits eaten fresh	Yes	TJ594
53.	Moraceae	*Ficus racemosa* L.	Ma Duea	Native	Tree	FT, VG	Ft	Fruit: Ripe fruits eaten fresh; unripe fruits consumed as fresh vegetables	Yes	TJ625
54.	Moraceae	*Streblus asper* Lour.	Khoi	Native	Tree	FT	Ft, Wp	Fruit: Ripe fruits eaten fresh; Whole Plant: Grown as an ornamental species	No	TJ657
55.	Ochnaceae	*Ochna integerrima* (Lour.) Merr.	Chang Nao	Native	Tree	MP	Rt, Wp	Whole plant: Grown as an ornamental species; Medicinal uses detailed in Table S1	No	TJ643
56.	Phyllanthaceae	*Antidesma puncticulatum* Miq.	Ma Mao	Native	Tree	FT	Ft	Fruit: Ripe fruits are consumed as fruit	Yes	TJ593
57.	Phyllanthaceae	*Phyllanthus androgynus* (L.) Chakrab. & N.P.Balakr.	Phak Wan	Native	Shrub	VG	Lv	Leaf: Used as an ingredient in culinary preparations	Yes	TJ648
58.	Poaceae	*Bambusa bambos* (L.) Voss	Phai	Native	Herb	VG	St	Shoot: Young shoots or bamboo shoots are used in cooking; commonly roasted or fermented for preservation	Yes	TJ595
59.	Primulaceae	*Embelia subcoriacea* (C.B.Clarke) Mez	Som Khi Mot	Native	Shrub	FT, VG	Ft, Lv	Fruit: Ripe fruits eaten fresh; Leaf: Consumed as fresh vegetables	No	TJ622
60.	Rhamnaceae	*Ziziphus oenopolia* (L.) Mill.	Nam Lep Maeo	Native	Shrub	FT	Ft	Fruit: Ripe fruits eaten fresh	No	TJ666
61.	Rubiaceae	*Canthium berberidifolium* E.T.Geddes	Ngiang Duk	Native	Shrub	FT	Ft	Fruit: Ripe fruits eaten fresh	No	TJ600
62.	Rubiaceae	*Hymenodictyon orixense* (Roxb.) Mabb.	Som Kob	Native	Tree	VG	Lv, St	Leaf, Shoot: Consumed as fresh vegetables or used as ingredients in cooking	No	TJ633
63.	Rubiaceae	*Paederia linearis* Hook.f.	Tod Mu Tod Ma	Native	Climber	VG	Lv	Leaf: Consumed raw as a fresh vegetable	No	TJ645
64.	Rutaceae	*Clausena wallichii* Oliv.	Song Fa	Native	Tree	MP	Rt	Medicinal uses detailed in Table S1	No	TJ607
65.	Rutaceae	*Harrisonia perforata* (Blanco) Merr.	Si Fan Khon Tha	Native	Shrub	MP	Rt	Medicinal uses detailed in Table S1	No	TJ630
66.	Rutaceae	*Micromelum minutum* (G.Forst.) Wight & Arn.	Sa Mat	Native	Tree	MP	Lv, Rt	Medicinal uses detailed in Table S1	No	TJ640
67.	Salicaceae	*Casearia grewiifolia* Vent.	Pha Sam	Native	Shrub	MP	Lv, Rt	Medicinal uses detailed in Table S1	No	TJ603
68.	Salicaceae	*Flacourtia indica* (Burm.f.) Merr.	Mak Ben	Native	Tree	FT	Ft	Fruit: Ripe fruits eaten fresh	Yes	TJ626
69.	Sapindaceae	*Lepisanthes rubiginosa* (Roxb.) Leenh.	Ma Huat Pa	Native	Shrub	FT	Ft	Fruit: Ripe fruits eaten fresh	No	TJ635
70.	Sapindaceae	*Nephelium hypoleucum* Kurz	Kho Laen	Native	Tree	FT	Ft	Fruit: Ripe fruits eaten fresh	Yes	TJ642
71.	Smilacaceae	*Smilax perfoliata* Lour.	Khreu Khueang	Native	Climber	VG	St	Shoot: Used as a vegetable ingredient in local dishes	Yes	TJ654
72.	Stemonaceae	*Stemona collinsiae* Craib	Non Tai Yak	Native	Herb	MP	Rt	Medicinal uses detailed in Table S1	No	TJ656
73.	Vitaceae	*Ampelocissus martini* Planch.	Mak I Koi	Native	Climber	FT	Ft	Fruit: Eaten fresh; sometimes pounded with unripe banana to reduce irritation	Yes	TJ591
74.	Vitaceae	*Causonis trifolia* (L.) Mabb. & J.Wen	Khreu Hun Pae	Native	Climber	VG	Ft	Fruit: Used as a cooking ingredient in savory dishes	No	TJ605
75.	Zingiberaceae	*Boesenbergia rotunda* (L.) Mansf.	Kra Chai Pa	Native	Herb	CF	Rz	Rhizome: Used as a spice and component of curry paste, adding aroma and pungency	Yes	TJ597
76.	Zingiberaceae	*Curcuma angustifolia* Roxb.	Kra Chiao Daeng	Native	Herb	VG	Ic	Inflorescence: Boiled and eaten as a side vegetable with chili paste	Yes	TJ613
77.	Zingiberaceae	*Kaempferia marginata* Carey ex Roscoe	Tub Mup	Native	Herb	VG	Lv	Leaf: Consumed raw or cooked as a vegetable	No	TJ634
78.	Zingiberaceae	*Zingiber zerumbet* (L.) Roscoe ex Sm.	Ka Thue	Native	Herb	VG	Lv	Leaf: Eaten raw or cooked as part of local cuisine	Yes	TJ665

Abbreviation. Used parts: Bk (bark), Ft (fruit), Hw (heart wood), Ic (inflorescence), La (latex), Lv (leaf), Rt (root), Rz (rhizome), St (stem or shoot), Tb (tuber), Wp (whole plant); Utilization: CF (condiments and flavoring), FT (fruit), MP (medicinal edible plants), SF (staple food), SW (sweets/desserts/snacks), VG (vegetable).

**Table 3:** Toxic or potentially harmful of wild edible plant species and their associated effects.

Scientific Name	Toxic Parts/Compounds	Effects/Precautions
*Ampelocissus martini* Planch.	Fruit	Excessive intake may irritate the throat
*Celastrus paniculatus* Willd.	Seeds	Inedible; causes throat irritation when consumed
*Cryptolepis buchananii* R.Br. ex Roem. & Schult.	Bioactive cardiac compounds	Stimulates cardiac function; should not be taken in high doses or for extended periods [29]
*Dioscorea hispida* Dennst.	Tuber containing dioscorine	Toxic if raw, depresses central nervous system; must be processed to remove toxin before consumption [30]
*Diospyros mollis* Griff.	Ripe fruit	Not suitable for children, pregnant women, or patients; may cause diarrhea, blurred vision, or blindness if overconsumed
*Gluta usitata* (Will.) Ding Hou	Latex containing urushiol	Causes severe skin irritation, itching, and blistering; can penetrate clothing; may lead to infection if scratched [31]

**Table 4:** Informant consensus factor (F_ic_) of wild edible plant species by the Tai Yoy Ethnic Group in Akat Amnuai District.

Therapeutic Categories	Number of Use Report (N_ur_)	Number of Taxa (N_t_)	F_ic_
Poisoning/Toxicology	23	1	1.000
Central Nervous System Disorders	56	2	0.982
Eye Disorders	32	2	0.968
Musculoskeletal Disorders	186	7	0.968
Gastrointestinal Disorders	379	18	0.955
Reproductive Disorders	85	5	0.952
Skin Disorders	140	8	0.950
Obstetrics, Gynaecology and Urinary Disorders	78	5	0.948
Infection/Immune Disorders	238	14	0.945
Blood Disorders	73	5	0.944
Respiratory Disorders	90	6	0.944

**Table 5:** Jaccard’s similarity index comparing the present study with surrounding regions.

Study Area	SN	CS	JI	SM	References
Akat Amnuai District, Sakon Nakhon Province	78	-	-	-	Present study
Don Pu Ta Forest, Phak Tob Village, Pla Lo Sub-district, Waritchaphum District, Sakon Nakhon Province	61	12	0.094	9.40	[10]
Don Pu Ta Forest, Ban Kut Haet, Kut Bak Sub-district, Kut Bak District, Sakon Nakhon Province	154	18	0.084	8.40	[32]
Ban Phue District, Udon Thani Province	243	12	0.039	3.90	[33]
Muang District, Kalasin Province	140	4	0.019	1.90	[8]
Huai Mek District, Kalasin Province	55	1	0.008	8.00	[34]
Khong Chai District, Kalasin Province	291	26	0.076	7.60	[35]

Abbreviation. SN (species number, CS (common species), JI (Jaccard index), SM (similarity (%)).

**Table 6:** Economic value of wild edible plant species traded in local markets, including plant parts for trade (PPT), price (THB/kg), monthly sales volume (kg) (MSV), availability (Months/Year) (AVL), and estimated average yearly income per trader (THB/a Trader) (AYI).

Scientific Name	PPT	Price (THB/kg) *		MSV	AVL	AYI
		**Max**	**Min**			
*Amorphophallus paeoniifolius* (Dennst.) Nicolson	Tuber	40	30	16.5	2	1155.00
*Ampelocissus martini* Planch.	Fruit	40	20	12.3	3	1107.00
*Antidesma puncticulatum* Miq.	Fruit	35	25	20.1	4	2412.00
*Artocarpus lacucha* Buch.-Ham.	Fruit	50	45	13.6	3	1938.00
*Bambusa bambos* (L.) Voss	Young shoot	35	30	21.3	4	2769.00
*Boesenbergia rotunda* (L.) Mansf.	Rhizome	50	40	15.5	4	2790.00
*Calamus viminalis* Willd.	Young shoot	100	80	8.9	3	2403.00
*Cratoxylum cochinchinense* (Lour.) Blume	Inflorescence	55	40	14.6	4	2774.00
*Cratoxylum formosum* (Jack) Benth. & Hook.f. ex Dyer	Inflorescence	55	40	14.5	4	2755.00
*Curcuma angustifolia* Roxb.	Inflorescence	100	80	16.3	3	4401.00
*Dioscorea hispida* Dennst.	Tuber	90	50	10.4	2	1456.00
*Ficus racemosa* L.	Fruit	30	20	5.6	3	420.00
*Flacourtia indica* (Burm.f.) Merr.	Fruit	80	60	10	3	2100.00
*Hellenia speciosa* (J.Koenig) S.R.Dutta	Inflorescence	40	35	11.8	4	1770.00
*Momordica cochinchinensis* (Lour.) Spreng.	Fruit	15	10	15.3	5	956.25
*Nephelium hypoleucum* Kurz	Fruit	30	20	17.2	4	1720.00
*Oroxylum indicum* (L.) Kurz	Fruit	35	25	18.4	3	1656.00
*Phyllanthus androgynus* (L.) Chakrab. & N.P.Balakr.	Leaf	160	140	22.6	3	10,170.00
*Senegalia rugata* (Lam.) Britton & Rose	Leaf	80	50	10.6	4	2756.00
*Smilax perfoliata* Lour.	Young shoot	50	40	10.1	4	1818.00
*Spondias pinnata* (L.f.) Kurz	Fruit	60	40	14.2	6	4260.00
*Tacca leontopetaloides* (L.) Kuntze	Tuber	150	100	9.8	3	3675.00
*Terminalia chebula* Retz.	Fruit	20	15	7.5	4	525.00
*Zingiber zerumbet* (L.) Roscoe ex Sm.	Inflorescence	40	30	8.6	4	1204.00

* Exchange rate—1 USD = 32.39 THB (as of 9 November 2025).

## Data Availability

The data presented in this study are available on request from the corresponding authors.

## Supplementary Materials

The following supporting information can be downloaded at https://www.mdpi.com/article/10.3390/biology15010015/s1, Table S1: Fidelity Level (%FL) of WEPs by the Tai Yoy Ethnic Group in Akat Amnuai District, Sakon Nakhon Province, Thailand; Table S2: Cultural Food Significance Index (CFSI) evaluation of wild edible plants by the Tai Yoy Ethnic Group in Akat Amnuai District, Sakon Nakhon Province, Thailand.
