# Supplementary material for: Utilization of Wild Edible Plants by the Tai Yoy Ethnic Group in Akat Amnuai District, Sakon Nakhon Province, Thailand
Source: Biology (Basel). 2025 Dec 20;15(1):15. doi: 10.3390/biology15010015 (PMC12784834; doi:10.3390/biology15010015)
Supplement: Supplementary file 1 [file biology-15-00015-s001.zip › biology-4009440-supplementary.pdf]

biology

MDPI

Supplementary

Utilization of Wild Edible Plants by the Tai Yoy Ethnic Group in Akat Amnuai District, Sakon Nakhon Province, Thailand

Table S1. Fidelity Level (%FL) of WEPs by the Tai Yoy Ethnic Group in Akat Amnuai District, Sakon Nakhon Province, Thailand.

No.	Scientific name	FL	UP	CoP	Preparation	RoA	Application	Therapeutic Categories
1.	*Achyranthes aspera* L.	40.00	St	Fresh	Boiled in water and the liquid portion filtered for drinking	Oral	Regulates menstruation, relieves mild urinary disorders, diuretic	Obstetrics, Gynaecology and Urinary Disorders
		35.00	Wp	Fresh	Boiled in water and the liquid portion filtered for drinking	Oral	Aids digestion	Gastrointestinal Disorders
		25.00	Lv	Fresh	Boiled in water and the liquid portion filtered for drinking	Oral	Treats throat ailments, relieves sore throat with nodules or lumps in the throat	Gastrointestinal Disorders
2.	*Amphineurion marginatum* (Roxb.) D.J.Middleton	42.50	Rt	Fresh	Boiled in water and the liquid portion filtered for drinking	Oral	Treats urinary tract disorders and regulates menstruation	Obstetrics, Gynaecology and Urinary Disorders
		40.00	St	Fresh	Boiled in water and the liquid portion filtered for drinking	Oral	Treats diarrhea	Gastrointestinal Disorders

 https://doi.org/10.3390/biology15010015

		17.50	Rt	Fresh	Boiled in water and the liquid portion filtered for drinking	Oral	Restorative, strengthens the body during convalescence	Infection/Immune Disorders
3.	*Biancaea sappan* (L.) Tod.	55.00	Hw	Dry	Sun-dried to reduce moisture, boiled in water, and the liquid portion strained for use	Oral	Consumed as a tonic to nourish the blood	Blood Disorders
		45.00	Hw	Dry	Sun-dried to reduce moisture, boiled in water, and the liquid portion strained for use	Oral	Treats diarrhea	Gastrointestinal Disorders
4.	*Capparis flavicans* Kurz	62.50	Hw	Dry	Sun-dried, ground into powder, and burned to produce smoke for inhalation	Nasal	Relieves dizziness	Central Nervous System Disorders
		37.50	Lv	Fresh	Consumed fresh	Oral	Galactagogue: beneficial for women with low milk production or who wish to stimulate lactation after childbirth	Obstetrics, Gynaecology and Urinary Disorders
5.	*Casearia grewiifolia* Vent.	37.50	Lv	Fresh	Boiled in water and the liquid portion filtered for drinking	Oral	Treats poisonous bites and febrile illnesses	Infection/Immune Disorders
		37.50	Rt	Fresh	Boiled in water and the liquid portion filtered for drinking	Oral	Relieves flatulence and treats diarrhea	Gastrointestinal Disorders
		25.00	Lv	Fresh	Boiled to extract oil, which is applied to wounds and infected skin	Dermal	Treats skin disorders, including itchy rashes with parasites such as ringworm, tinea, and scabies	Skin Disorders

6.	*Cassytha filiformis* L.	40.00	St	Fresh	Boiled in water and the liquid portion filtered for drinking	Oral	Treats common cold and febrile illnesses	Infection/Immune Disorders
		25.00	St	Fresh	Boiled in water and the liquid portion filtered for drinking	Oral	Diuretic; treats urinary stones, urinary tract infections, and inflammation of the urinary tract	Obstetrics, Gynaecology and Urinary Disorders
		35.00	St	Fresh	Pounded into a paste and applied to the affected skin area	Oral	Treats wounds from falls and controls bleeding	Skin Disorders
7..	*Celastrus paniculatus* Willd.	77.50	Lv	Fresh	Leaves are boiled or squeezed for juice and taken orally	Oral	Used as a nervous system stimulant	Central Nervous System Disorders
		22.50	Hw	Fresh	Boiled in water and the liquid portion filtered for drinking	Oral	Used for tuberculosis treatment	Infection/Immune Disorders
8.	*Clausena wallichii* Oliv.	50.00	Rt	Fresh	Boiled in water and the liquid portion filtered for drinking	Oral	Used to relieve flatulence and abdominal bloating	Gastrointestinal Disorders
		25.00	Rt	Fresh	Boiled in water and the liquid portion filtered for drinking	Oral	Used for treating fever and headache	Infection/Immune Disorders
9.	*Connarus semidecandrus* Jack	37.50	Rt	Fresh	Boiled in water and the liquid portion filtered for drinking	Oral	Used for treating fever	Infection/Immune Disorders
		32.50	Wp	Fresh	Boiled in water and the liquid portion filtered for drinking	Oral	Used as a laxative	Gastrointestinal Disorders
		30.00	Bk	Fresh	Boiled in water and the liquid portion filtered for drinking	Oral	Used for treating abdominal pain	Gastrointestinal Disorders

10.	*Cryptolepis buchananii* R.Br. ex Roem. & Schult.	65.00	St	Dry	The dried plant material is ground into powder and mixed with rice whisky before being taken orally	Oral	Used for treating bruises and contusions	Musculoskeletal Disorders
		35.00	Lv	Fresh	Pounded and wrapped in cloth to make a herbal compress, applied on tendons and muscles to relieve pain, stiffness, and soreness	Dermal	Used to relieve body aches, muscle stiffness, and improve tendon flexibility	Musculoskeletal Disorders
11.	*Curculigo latifolia* Dryand. ex W.T.Aiton	50.00	Rt	Fresh	Boiled in water and the liquid portion filtered for drinking	Oral	Used to promote physical strength and enhance overall well-being	Musculoskeletal Disorders
		50.00	Lv	Dry	Sun-dried to reduce moisture, boiled in water, and the liquid portion strained for use	Oral	Employed in folk medicine to treat coughs and throat inflammation	Reproductive Disorders
12.	*Cynanchum pulchellum* (Wall.) Liede & Khanum	57.50	Rt	Fresh	Boiled in water and the liquid portion filtered for drinking	Oral	Used as an antidote; induces vomiting and neutralizes various poisons	Poisoning/Toxicology
		42.50	Rt	Dry	The dried roots are ground into powder and taken orally as medicine	Oral	Traditionally used to treat various eye ailments, including redness, inflammation, watering eyes, blurred vision, and eye opacity	Eye Disorders

13.	*Diospyros ehretioides* Wall. ex G.Don	65.00	Rt	Dry	Sun-dried to reduce moisture, boiled in water, and the liquid portion strained for use	Oral	Used for treating fever	Infection/Immune Disorders
		35.00	Rt	Dry	Sun-dried to reduce moisture, boiled in water, and the liquid portion strained for use	Oral	Used to treat tuberculosis	Infection/Immune Disorders
14.	*Diospyros mollis* Griff.	52.50	Ft	Fresh	The unripe fruits are pounded and squeezed to extract the juice, which is mixed with fresh coconut cream and taken immediately before breakfast. The preparation must be freshly made, as storing it may cause toxicity	Oral	Used as an anthelmintic for roundworms, hookworms, and threadworms	Infection/Immune Disorders
		47.50	Bk	Fresh	Boiled in water and the liquid portion filtered for drinking	Oral	Used to stimulate appetite and as an expectorant	Gastrointestinal Disorders
15.	*Dipterocarpus obtusifolius* Teijsm. ex Miq.	72.50	Lv	Fresh	The plant part is boiled with salt, and the decoction is used as a mouth rinse	Oral	Used to relieve toothache and strengthen loose teeth	Gastrointestinal Disorders
		27.50	Bk	Fresh	Boiled in water and the liquid portion filtered for drinking	Oral	Treats diarrhea	Gastrointestinal Disorders
16.	*Dipterocarpus tuberculatus* Roxb.	55.00	Lv	Fresh	The plant part is boiled with salt, and the decoction is used as a mouth rinse	Oral	Used to relieve toothache and strengthen loose teeth	Gastrointestinal Disorders

		45.00	Lv	Fresh	Pounded and applied to the affected area	Dermal	Treats insect bites	Skin Disorders
17.	*Elephantopus scaber* L.	50.00	Rt	Fresh	Boiled in water and the liquid portion filtered for drinking	Oral	Used as an aphrodisiac; enhances sexual performance	Reproductive Disorders
		27.50	Rt	Fresh	Macerated in liquor, taken orally	Oral	Used as a tonic to relieve body pain and fatigue	Musculoskeletal Disorders
		22.50	Lv	Fresh	Boiled with coconut oil and applied to wounds	Dermal	Used to treat wounds and skin diseases	Skin Disorders
18.	*Erythroxylum cuneatum* (Miq.) Kurz	67.50	Bk	Fresh	Boiled in water and the liquid portion filtered for drinking	Oral	Used to treat tendon disorders, relieve muscular pain, and reduce numbness of the hands and feet	Musculoskeletal Disorders
		32.50	Rt	Dry	Sun-dried to reduce moisture, boiled in water, and the liquid portion strained for use	Oral	Used as a general tonic to promote overall health	Musculoskeletal Disorders
19.	*Garcinia cowa* Roxb. Ex Choisy	50.00	Rt	Fresh	Boiled in water and the liquid portion filtered for drinking	Oral	Used as a laxative to relieve constipation	Gastrointestinal Disorders
		50.00	Lv	Fresh	Boiled in water and the liquid portion filtered for drinking	Oral	Used for treating fever	Infection/Immune Disorders
20.	*Gluta usitata* (Will.) Ding Hou	60.00	Bk	Dry	Sun-dried to reduce moisture, boiled in water, and the liquid portion strained for use	Oral	Treats diarrhea	Gastrointestinal Disorders

		40.00	St	Fresh	Boiled in water and the liquid portion filtered for drinking	Oral	Used to treat hematemesis (vomiting of blood)	Gastrointestinal Disorders
21.	*Harrisonia perforata* (Blanco) Merr.	45.00	Rt	Fresh	Boiled in water and the liquid portion filtered for drinking	Oral	Used for treating fever	Infection/Immune Disorders
		37.50	Rt	Fresh	Boiled in water and the liquid portion filtered for drinking	Oral	Treats eye pain and inflammation	Eye Disorders
		17.50	Rt	Fresh	Boiled in water and the liquid portion filtered for drinking	Oral	Promotes blood circulation	Blood Disorders
22.	*Hellenia speciosa* (J.Koenig) S.R.Dutta	50.00	Rz	Fresh	Boiled in water and the liquid portion filtered for drinking	Oral	Treats urinary tract infections; nourishes the uterus	Obstetrics, Gynaecology and Urinary Disorders
		37.50	Rz	Dry	Sun-dried to reduce moisture, boiled in water, and the liquid portion strained for use	Oral	Relieves cough and expels phlegm	Respiratory Disorders
		12.50	Rz	Fresh	Pounded into a fine paste and applied to the umbilicus	Dermal	Used for treating abdominal dropsy (ascites)	Gastrointestinal Disorders
23.	*Huberantha cerasoides* (Roxb.) Chaowasku	37.50	Rt	Fresh	Boiled in water and the liquid portion filtered for drinking	Oral	Used for treating fever	Infection/Immune Disorders
		37.50	Rt	Fresh	Boiled in water and the liquid portion filtered for drinking	Oral	Used as an aphrodisiac and tonic to enhance male sexual performance, relieve muscle tension, and rejuvenate the body	Reproductive Disorders

		25.00	Lv	Fresh	Pounded and applied to the affected area	Dermal	Used to treat abscesses, relieve pain, and reduce inflammation	Skin Disorders
24.	*Micromelum minutum* (G.Forst.) Wight & Arn.	47.50	Lv	Fresh	Boiled in water and the liquid portion filtered for drinking	Oral	Used as an aphrodisiac and tonic to enhance male sexual performance, relieve muscle tension, and rejuvenate the body	Reproductive Disorders
		27.50	Rt	Fresh	Boiled in water and the liquid portion filtered for drinking	Oral	Promotes blood circulation	Blood Disorders
		25.00	Lv	Fresh	Boiled in water and the liquid portion filtered for drinking	Oral	Used as a carminative to relieve flatulence and abdominal discomfort	Gastrointestinal Disorders
25.	*Ochna integerrima* (Lour.) Merr.	70.00	Rt	Fresh	Macerated in liquor; taken orally	Oral	Used as a tonic to strengthen the body, relieve fatigue and muscular pain, and treat kasai (general weakness)	Musculoskeletal Disorders
		30.00	Rt	Fresh	Boiled in water and the liquid portion filtered for drinking	Oral	Used as a tonic to strengthen and improve the digestive system	Gastrointestinal Disorders
26.	*Peltophorum dasyrhachis* (Miq.) Kurz	55.00	Bk	Fresh	Boiled in water and the liquid portion filtered for drinking	Oral	Relieves cough and expels phlegm	Respiratory Disorders

		45.00	Bk	Fresh	Boiled in water and the liquid portion filtered for drinking	Oral	Used to treat blood-related disorders and improve blood circulation	Blood Disorders
27.	*Phanera sirindhorniae* (K.Larsen & S.S.Larsen) Mackinder & R.Clark	62.50	St	Fresh	Boiled in water and used for bathing	Dermal	Used to treat rashes, itching, and pradong (chronic skin eruptions)	Skin Disorders
		37.50	St	Fresh	Boiled in water and the liquid portion filtered for drinking	Oral	Used as a blood tonic to nourish and improve blood quality and circulation	Blood Disorders
28.	*Pueraria mirifica* Airy Shaw & Suvat.	72.50	Tb	Dry	Dried, powdered, mixed with honey, and formed into pills	Oral	Used as a tonic and rejuvenating medicine to promote vitality and longevity in the elderly	Musculoskeletal Disorders
		27.50	Tb	Dry	Dried, powdered, mixed with honey, and formed into pills	Oral	Used as an aphrodisiac to increase sexual desire, improve blood circulation to the reproductive organs and uterus, and enhance reproductive vitality	Reproductive Disorders
29.	*Rourea stenopetala* (Griff.) Hook.f.	62.50	Ft	Dry	The plant material is sun-dried, ground into powder, and infused in warm water for oral consumption	Oral	Used as an expectorant to relieve cough and expel phlegm	Respiratory Disorders
		37.50	Ft	Dry	The plant material is sun-dried, ground into powder, and infused	Oral	Used for treating fever	Infection/Immune Disorders

					in warm water for oral consumption			
30.	*Senegalia pennata* (L.) Maslin	67.50	Ft	Fresh	Eaten fresh	Oral	Used as a laxative	Gastrointestinal Disorders
		20.00	Ft	Fresh	Eaten fresh	Oral	Used as an expectorant and for relieving cough	Respiratory Disorders
		12.50	Ft	Fresh	Eaten fresh	Oral	Used for relieving asthma	Respiratory Disorders
31.	*Stemona collinsiae* Craib	55.00	Rt	Fresh	Boiled in water and the liquid portion filtered for drinking	Oral	Used to expel intestinal worms	Infection/Immune Disorders
		35.00	Rt	Fresh	Boiled in water and the liquid portion filtered for drinking	Oral	Used for treating skin diseases and blood impurities	Skin Disorders
		10.00	Rt	Fresh	Boiled in water and the liquid portion filtered for drinking	Oral	Used as an expectorant to relieve cough and expel phlegm	Respiratory Disorders
32.	*Streptocaulon juventas* (Lour.) Merr.	45.00	Rt	Fresh	Boiled in water and the liquid portion filtered for drinking	Oral	Used as a tonic and rejuvenating medicine to promote vitality	Musculoskeletal Disorders
		37.50	Rt	Fresh	Boiled in water and the liquid portion filtered for drinking	Oral	Used for treating fever	Infection/Immune Disorders
		17.50	Rt	Fresh	Boiled in water and the liquid portion filtered for drinking	Oral	Remedy for abdominal pain, gastric disorders, and diarrhea	Gastrointestinal Disorders

33.	*Suregada multiflora* (A.Juss.) Baill.	72.50	Bk	Fresh	Boiled in water and the liquid portion filtered for drinking	Oral	Used to treat skin diseases, eczema, and fungal infections	Skin Disorders
		27.50	Rt	Fresh	Boiled in water and the liquid portion filtered for drinking	Oral	Treats pradong (chronic skin eruptions)	Skin Disorders
34.	*Terminalia chebula* Retz.	67.50	Ft	Fresh	Eaten fresh	Oral	Used as a laxative	Gastrointestinal Disorders
		32.50	Ft	Fresh	Eaten fresh	Oral	Used to relieve flatulence and abdominal pain	Gastrointestinal Disorders
35.	*Terminalia elliptica* Willd.	47.50	Bk	Fresh	Boiled in water and the liquid portion filtered for drinking	Oral	Used to treat diarrhea	Gastrointestinal Disorders
		27.50	Bk	Fresh	Boiled in water and the liquid portion filtered for drinking	Oral	Used as an expectorant to relieve cough and expel phlegm	Respiratory Disorders

Abbreviation. UP (used parts): Bk (bark), Ft (fruit), Hw (heart wood), Lv (leaf), Rt (root), Rz (rhizome), St (stem or shoot), Tb (tuber), Wp (whole plant); CoP (condition of plants used); RoA (route of administration).

Table S2. Cultural Food Significance Index (CFSI) evaluation of wild edible plants by the Tai Yoy Ethnic Group in Akat Amnuai District, Sakon Nakhon Province, Thailand.

Scientific name	QI	AI	FUI	PUI	MFFI	TSAI	FMRI	CFSI
Hellenia speciosa (J.Koenig) S.R.Dutta	23	4	3	4.75	1	6.5	5	426.08
Senegalia pennata (L.) Maslin	26	4	3	3	1.5	9	3	379.08
Urceola polymorpha (Pierre ex Spire) D.J.Middleton & Livsh.	23	4	3	3	1.5	9	3	335.34
Momordica cochinchinensis (Lour.) Spreng.	27	4	3	4.25	1	7.5	3	309.83
Spondias pinnata (L.f.) Kurz	21	4	5	1.5	1.5	9	3	255.15
Bambusa bambos (L.) Voss	28	4	5	1	1.5	10	3	252.00

Senna timoriensis (DC.) H.S.Irwin & Barneby	25	4	4	1.5	1.5	9	3	243.00
Dioscorea hispida Dennst.	24	4	3	1.5	1.5	10	3	194.40
Calamus viminalis Willd.	26	4	3	1.5	1.5	9	3	189.54
Careya arborea Roxb.	22	4	4	1.5	1.5	7.5	3	178.20
Tacca leontopetaloides (L.) Kuntze	21	4	3	1.5	1.5	10	3	170.10
Phyllanthus androgynus (L.) Chakrab. & N.P.Balakr.	29	4	2	1.5	1.5	10	3	156.60
Oroxylum indicum (L.) Kurz	19	4	3	2.25	1.5	6.5	3	150.05
Kaempferia marginata Carey ex Roscoe	24	4	3	1.5	1.5	7.5	3	145.80
Streblus asper Lour.	20	4	4	4.5	0.5	6.5	3	140.40
Zingiber zerumbet (L.) Roscoe ex Sm.	19	4	3	1.5	1.5	7.5	3	115.43
Curcuma angustifolia Roxb.	28	4	3	0.75	1.5	10	3	113.40
Piliostigma malabaricum (Roxb.) Benth.	18	4	3	1.5	1.5	7.5	3	109.35
Phanera sirindhorniae (K.Larsen & S.S.Larsen) Mackinder & R.Clark	16	3	2	4	1	5.5	5	105.60
Amorphophallus paeoniifolius (Dennst.) Nicolson	25	4	3	1.25	1	9	3	101.25
Cratoxylum formosum (Jack) Benth. & Hook.f. ex Dyer	25	4	3	0.75	1.5	10	3	101.25
Cratoxylum cochinchinense (Lour.) Blume	24	4	3	0.75	1.5	10	3	97.20
Smilax perfoliata Lour.	27	4	2	1	1.5	10	3	97.20
Peltophorum dasyrhachis (Miq.) Kurz	18	3	2	4	1	5.5	4	95.04
Boesenbergia rotunda (L.) Mansf.	18	4	3	1	1.5	9	3	87.48
Antidesma puncticulatum Miq.	25	4	4	1.5	0.5	9	3	81.00
Ficus racemosa L.	23	3	2	1.5	1.5	7.5	3	69.86
Memecylon edule Roxb.	16	3	1	5.5	1.5	5.5	3	65.34
Pueraria mirifica Airy Shaw & Suvat.	24	4	2	1.5	1	5.5	4	63.36
Dipterocarpus obtusifolius Teijsm. ex Miq.	18	3	2	3.5	1	4	4	60.48
Clausena wallichii Oliv.	17	3	3	1.5	1	6.5	4	59.67
Biancaea sappan (L.) Tod.	17	3	2	2	1	5.5	5	56.10
Paederia linearis Hook.f.	18	3	2	1.5	1.5	7.5	3	54.68
Connarus semidecandrus Jack	9	2	2	5.5	1	5.5	4	43.56
Amphineurion marginatum (Roxb.) D.J.Middleton	13	2	2	2.75	1	5.5	5	39.33

Ziziphus oenopolia (L.) Mill.	24	4	2	1.5	0.5	9	3	38.88
Artocarpus lacucha Buch.-Ham.	24	3	2	1.5	0.5	10	3	32.40
Nephelium hypoleucum Kurz	20	4	2	1.5	0.5	9	3	32.40
Hymenodictyon orixense (Roxb.) Mabb.	13	2	2	2.5	1.5	5.5	3	32.18
Achyranthes aspera L.	9	4	1	3.75	1	5.5	4	29.70
Canthium berberidifolium E.T.Geddes	22	4	2	1.5	0.5	7.5	3	29.70
Lepisanthes rubiginosa (Roxb.) Leenh.	19	4	2	1.5	0.5	7.5	3	25.65
Dipterocarpus tuberculatus Roxb.	17	3	2	1.5	1	4	4	24.48
Flacourtia indica (Burm.f.) Merr.	18	4	2	1.5	0.5	7.5	3	24.30
Cryptolepis buchananii R.Br. ex Roem. & Schult.	8	2	2	2.75	1	5.5	5	24.20
Terminalia chebula Retz.	15	4	2	1.5	0.5	7.5	3	20.25
Mangifera caloneura Kurz	12	3	1	1.5	1.5	7.5	3	18.23
Gluta usitata (Will.) Ding Hou	11	2	1	3	1	5.5	5	18.15
Rourea stenopetala (Griff.) Hook.f.	11	2	2	1.5	1	5.5	5	18.15
Gnetum gnemon L.	8	3	2	1.5	1.5	5.5	3	17.82
Terminalia elliptica Willd.	10	2	2	2	1	5.5	4	17.60
Ochna integerrima (Lour.) Merr.	14	1	1	4.5	1	6.5	4	16.38
Huberantha cerasoides (Roxb.) Chaowasku	10	2	2	4.5	0.5	5.5	3	14.85
Cynanchum pulchellum (Wall.) Liede & Khanum	9	2	2	1.5	1	5.5	5	14.85
Canarium subulatum Guillaumin	9	4	2	1.5	0.5	9	3	14.58
Diospyros mollis Griff.	8	2	2	2.5	1	4	4	12.80
Streptocaulon juventas (Lour.) Merr.	5	4	2	1.5	1	4	5	12.00
Fernandoa adenophylla (Wall. ex G.Don) Steenis	9	3	2	0.75	1.5	6.5	3	11.85
Curculigo latifolia Dryand. ex W.T.Aiton	6	2	1	3	1	6.5	5	11.70
Garcinia cowa Roxb. Ex Choisy	8	1	1	6.5	1	5.5	4	11.44
Embelia subcoriacea (C.B.Clarke) Mez	5	2	2	3	1.5	4	3	10.80
Markhamia stipulata (Wall.) Seem.	8	3	2	0.75	1.5	6.5	3	10.53
Diospyros ehretioides Wall. ex G.Don	7	2	2	1.5	1	5.5	4	9.24
Elephantopus scaber L.	5	2	1	3	1	5.5	5	8.25

Casearia grewiifolia Vent.	4	2	1	3	1	5.5	5	6.60
Erythroxylum cuneatum (Miq.) Kurz	4	2	2	2.5	1	4	4	6.40
Ampelocissus martini Planch.	14	3	1	1.5	0.5	6.5	3	6.14
Suregada multiflora (A.Juss.) Baill.	7	2	1	2.5	1	4	4	5.60
Strophioblachia fimbricalyx Boerl.	5	2	1	2.25	1.5	4	4	5.40
Micromelum minutum (G.Forst.) Wight & Arn.	3	2	1	3	1	5.5	5	4.95
Harrisonia perforata (Blanco) Merr.	5	1	2	1.5	1	6.5	5	4.88
Causonis trifolia (L.) Mabb. & J.Wen	9	2	1	1.5	1.5	4	3	4.86
Capparis flavicans Kurz	4	1	1	4.75	1	5.5	4	4.18
Celastrus paniculatus Willd.	5	1	1	2.75	1	5.5	5	3.78
Stemona collinsiae Craib	4	3	1	1.5	1	4	5	3.60
Dillenia hookeri Pierre	6	2	2	3	0.5	3	3	3.24
Cassytha filiformis L.	4	2	1	1	1	5.5	5	2.20
Litsea glutinosa (Lour.) C.B.Rob.	5	3	1	1.5	0.5	5.5	3	1.86

Abbreviation. QI (Quotation Index); AI (Availability Index); FUI (Frequency of Utilization Index); PUI (Parts Used Index); MFFI (Multifunctional Food Use Index); TSAI (Taste Score Appreciation Index); FMRI (Food-Medicinal Role Index); CFSI (Cultural Food Significance Index).
